# Variable-Internal-Stores models of microbial growth and metabolism with dynamic allocation of cellular resources

**DOI:** 10.1007/s00285-016-1030-4

**Published:** 2016-06-06

**Authors:** Olga A. Nev, Hugo A. van den Berg

**Affiliations:** 1 Warwick Analytical Sciences Centre, University of Warwick, Coventry, CV4 7AL UK; 2 University of Warwick, Coventry, CV4 7AL UK

**Keywords:** Microbial growth, Multiple nutrient limitation, Cellular resource allocation, Physiological regulation, 92B05, 34D20

## Abstract

Variable-Internal-Stores models of microbial metabolism and growth have proven to be invaluable in accounting for changes in cellular composition as microbial cells adapt to varying conditions of nutrient availability. Here, such a model is extended with explicit allocation of molecular building blocks among various types of catalytic machinery. Such an extension allows a reconstruction of the regulatory rules employed by the cell as it adapts its physiology to changing environmental conditions. Moreover, the extension proposed here creates a link between classic models of microbial growth and analyses based on detailed transcriptomics and proteomics data sets. We ascertain the compatibility between the extended Variable-Internal-Stores model and the classic models, demonstrate its behaviour by means of simulations, and provide a detailed treatment of the uniqueness and the stability of its equilibrium point as a function of the availabilities of the various nutrients.

## Introduction

Models of bacterial growth can be written as ${\dot{W}}=\mu ({\mathbf {x}},{\mathbf {u}})W$, where $W\in {\mathbb {R}}^+$ is a suitable measure of biomass, ${\mathbf {x}}\in {\mathbb {R}}^p$ represents the internal state, ${\mathbf {u}}\in {\mathbb {R}}^q$ represents external conditions that impinge on ${\dot{W}}$, and the dot indicates differentiation with respect to time (Dawes 1989). A basic model in this class specifies $\mu ([\text {N}])={\widehat{\mu }}\left( 1+K_S/[\text {N}]\right) ^{-1}$, where $[\text {N}]$ is the ambient concentration of the limiting nutrient and ${\widehat{\mu }}$ and $K_S$ are positive parameters (Monod 1949). Here, $p=0$ and $q=1$: there are no state variables other than *W* and there is a single environmental variable on which the specific growth rate $\mu$ depends. We allow $[\text {N}]$ to vary in time so that $W(t)=W_0\exp \left\{ \int _{0}^{t}{\mu \left( [\text {N}](\tau )\right) d\tau }\right\}$. One way to extend this model to $q>1$, but still with $p=0$, is to posit a multiplicative form $\mu (u_1,u_2,\dots )={\widehat{\mu }}f_1(u_1)f_2(u_2)\cdots$ (Gottschal 1992; de Wit et al. 1995), where the $u_1, u_2,\dots$ are salient environmental factors (such as levels of light, nutrients, redox substrates) and the $f_1, f_2,\dots$ are appropriate functions ${\mathbb {R}}^+\mapsto [0,1]$ that express how these factors affect growth.

Regarding models with $p>0$, one might decide to account explicitly for the position and movement of every molecule inside the cell ($p\sim 10^{8}$) or at least for the concentrations of all molecular species ($p\sim 10^{3}$–$10^5$, depending on how species are defined; Ederer et al. 2014). The cases $p=0$ and $p\sim 10^{8}$ represent opposite ends of a spectrum; which of the two is more suitable depends on the available information as well as the purpose at hand; we are often interested in the rates at which other compounds besides the biomass are being produced, and this typically requires physiological structuring beyond $p=0$. Our point of departure is a class of models that lies at a mid-way point on this spectrum, with *p* somewhere between 1 and a few dozen, known as Variable-Internal-Stores (VIS) models (Williams 1967; Droop 1968; Grover 1991). Taking into account internal stores, which in prokaryotes occur as metabolite pools, reserve compounds, and elemental inclusions (Beveridge 1989; Preiss 1989; Neidhardt et al. 1990), allows an accurate description of the rates of resource consumption and bioproduction yields (Dawes 1989).

In addition to VIS, we consider variations in the distribution of molecular building blocks among various types of molecular machinery (Bleecken 1988; van den Berg 2001). It is *a priori* likely that this allocation of building blocks is an important dynamic variable (Li et al. 2014); expression of genes is modulated, in prokaryotes as in eukaryotes, in response to changes in external conditions as well as in the status of internal availability of substrate (Neidhardt et al. 1990), and changes in the gene expression profile are reflected in corresponding changes in the relative rates at which molecular building blocks are incorporated into molecular machinery (Kramer et al. 2010). Furthermore, in prokaryotes, the ability to adjust resource re-allocation among catalytic machinery has been shown to be an evolutionarily relevant trait, at least for certain kinds of ecological life history (van den Berg et al. 1998). Finally, VIS-plus-reallocation models should enable the reconstruction of regulatory rules that drive this re-allocation by combining stoichiometric constraints with observations of transient behaviour following changes in environmental conditions. For instance, in a continuous-culture system, such perturbations can be imposed by the experimenter and the response measured in terms of cellular composition, cellular density, as well as consumption and production of relevant chemical species (e.g., de Wit et al. 1995).

The present paper describes the basic structure of VIS-plus-reallocation models, taking care to distinguish fundamental stoichiometric principles such as mass conservation from the constitutive relations that express the regulatory rules. We discuss the compatibility of this new class of models with well-established empirical laws in microbial growth and metabolism, as well as the observability of these constitutive relations. Moreover, we prove the uniqueness and stability of the equilibrium point under a reasonable assumption on the general appearance of the constitutive relations.

## Variable internal stores plus dynamic allocation theory

**Fig. 1:**
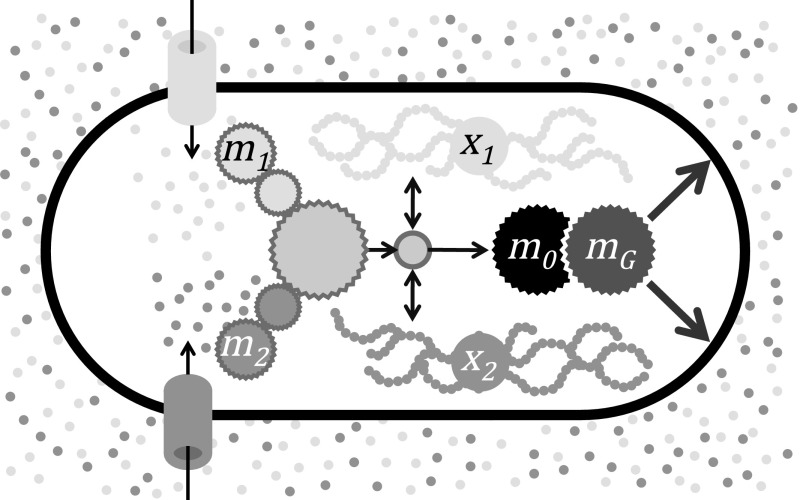
Schematic representation of the model described by the system (8) for the case $n=2$. Two types of nutrients are assimilated by dedicated pathways ($m_1$ and $m_2$) that feed into core metabolism from which building blocks are sluiced to machinery synthesis ($m_0$) and growth ($m_G$). Core metabolism also exchanges molecular building blocks with reserves ($x_1$ and $x_2$)

The model consists of stoichiometric equations, which are based on standard chemical conservation principles, presented in Sect. 2.1, and constitutive relations, which express specific assumptions regarding the regulatory control pathways; one simple choice is discussed in Sect. 2.2. A schematic representation of the model (for $n=2$) is given in Fig. 1. Notation is summarised in Table 1, and key simplifying assumptions are summarised in Table 2.

**Table 1:** Notation employed in the equations describing the model

Symbol	Biological interpretation	Units
State unscaled variables		
$M_i$	C-molar amount of *i*-type molecular machinery	Moles of carbon
$X_j$	Molar amount of the primary element $X_j$ in a reserve *j*	Moles of the primary element in a reserve
*W*	C-molar amount of the structural component	Moles of carbon
${\widetilde{\mu }}$	Specific growth rate	Per unit of time
State scaled variables		
$m_i$	Density of *i*-type molecular machinery	Dimensionless
$x_j$	Density of a reserve *j*	Dimensionless
$\mu$	Specific growth rate	Dimensionless
Unscaled stoichiometric coefficients		
${\widetilde{\phi }_i}$	The rate of production of the machinery of type *i*	Units of $M_i$ per unit of $M_0$ per unit of time
${\widetilde{\psi }_{ji}}$	The gain of reserve *j* per unit machinery of type *i*	Units of $X_j$ per unit of $M_i$ per unit of time
${\widetilde{\sigma }_{jW}}$	The loss of reserve *j* for growth	Units of $X_j$ per unit of *W*
${\widetilde{\sigma }_{ji}}$	The loss of reserve *j* for synthesis of the machinery of type *i*	Units of $X_j$ per unit of $M_i$
${\widetilde{\psi }_W}$	The rate of production of the structural component	Units of *W* per unit of $M_G$ per unit of time
Scaled stoichiometric coefficients		
$\psi _{j i}$	The gain of reserve *j* per unit machinery of type *i*	Dimensionless
$\sigma _{j i}$	The loss of reserve *j* for synthesis of the machinery of type *i*	Dimensionless
$\psi _W$	The rate of production of the structural component	Dimensionless
Constitutive relationships		
$\alpha _i$	Portion of the zero machinery devoted to the synthesis of machinery of type *i*	Dimensionless
${\widetilde{r}_i}$	Concentration of translationally active mRNA for the machinery of type *i*	Units of concentration
$r_i$	Scaled variable for the ${\widetilde{r}_i}$	Dimensionless
*K*	Slope of the increasing part of the piecewise function $r_G$	Dimensionless
$\epsilon$	Defines the interval on the abscissa for the increasing part of the piecewise function $r_G$	Dimensionless
Miscellany		
${\widehat{\widetilde{\phi _i}}}$	Maximum rate of the flux through the assimilatory machinery of type *i*	Units of nutrient per unit of $M_i$ per unit of time
$f_i$	Defines the ambient conditions for the nutrient *i*	Dimensionless
${\mathbf {R}}$	Chemical composition of the reserves as an $n\times n$ matrix	
${\mathbf {N}}$	Chemical composition of the nutrients as an $n\times n$ matrix	
$\gamma _{j i}$	(*j*, *i*)th element of ${\mathbf {R}}^{-1}\cdot {\mathbf {N}}$	Dimensionless
${\widehat{m}}$	Scaling parameter for $M_0/W$	Units of $M_0$ per unit of *W*

for all subscripts $i, j\; : i\in \{0,1,\dots ,n,G\}, j\in \{1,\dots ,n\}$

**Table 2:** Assumptions used in the analysis of the model

Assumption	Biological interpretation
$\sigma _{j i} = \sigma _j$ for all *i*	Amounts of reserves expended on the synthesis of different types of machineries are the same
$\psi _{j i} = 0$ whenever $j\ne i$	The elemental ratios of the reserves are identical to the elemental ratios of the nutrients
${\widetilde{r}_0}$ is constant	Constitutive expression (housekeeping mRNA)

for all subscripts $i, j\; : i\in \{0,1,\dots ,n,G\}, j\in \{1,\dots ,n\}$

### Stoichiometric equations

#### Basic definitions and dynamics

The bacterial cell is conceptually divided into several components, comprising molecular machinery, reserve compounds, and a structural component. The latter includes the cell envelope, genetic material, and core metabolites, small molecules that occur as intermediates of catabolic and anabolic pathways that are maintained at appropriate cellular concentrations by mechanisms not represented explicitly in the model. The C-molar amount of the structural component will be denoted as *W*.

Molecular machinery is divided into $n+2$ components, where *n* is the number of chemical species of nutrient for which we wish to account (this choice is informed by available data as well as the envisaged application of the theory). Components 1 through *n* represent the apparatus dedicated to the assimilation of the corresponding nutrients (transporters, binding proteins), in addition to the catalytic machinery that transforms these nutrients into core metabolites. Component 0 is the machinery required to synthesise machinery. Component $n+1$, which will be given the subscript G, represents machinery devoted to growth, that is, the synthesis of the cell envelope and duplication of the genome. The C-molar amounts of these $n+2$ types of machinery will be denoted as $M_i$.

Reserve components correspond to storage of nutrients which is mobilised by the cell to replenish the central pools of core metabolites. We allow for *n* distinct types of such variable internal stores. Certain reserves can be quantified in terms of C-moles, such as organic polymers such as poly-$\beta$-hydroxybutyrate, saccharides, as well as storage proteins, whereas others, such as sulphur globules and polyphosphate inclusions that contain no carbon (Preiss 1989), are expressed in terms of molar amounts of the primary element $\mathrm{X}_j$. These $\mathrm{X}_j$-molar amounts (where $\mathrm{X}_j$ is possibly but not necessarily C) will be denoted as $X_j$.

Although machinery is a heterogeneous assembly of proteins, nucleic acids, and co-factors (Neidhardt et al. 1990), it is nonetheless reasonable to assume that its chemical composition exhibits negligible fluctuations about the average typical of each kind of machinery. The dynamics of each component can then simply be written as follows:

1 $$\begin{aligned} {\dot{M}}_i=\alpha _i M_0 {\widetilde{\phi }}_i, \end{aligned}$$

where $i\in \{0,1,\dots ,n,G\}$, $\alpha _i$ is an allocation coefficient indicating which portion of the zero machinery is devoted to the synthesis of machinery of type *i*, and ${\widetilde{\phi }}_i$ is a stoichiometric coefficient. Parameters are indicated with a tilde to signify that they are dimensional; this allows the use of the same symbols when the model is rendered dimensionless (Sect. 2.1.2). Being a fraction, $\alpha _i$ is non-negative and subject to the constraint

2 $$\begin{aligned} \sum _{i\in \{0,1,\dots ,n,G\}}{\alpha _i}=1. \end{aligned}$$

A unit of zero machinery spends a fraction $\alpha _i$ of its time producing *i*-type machinery. Thus, when $\alpha _i=1$, every unit of time, ${\widetilde{\phi }}_i$ units of machinery of type *i* are being produced per unit of zero machinery. The reserve components change according to the balance of uptake and expenditures (Dawes 1989):

3 $$\begin{aligned} {\dot{X}}_j=\sum _{i=1}^{n}{{\widetilde{\psi }}_{ji} M_i}-{\widetilde{\sigma }}_{jW}{\dot{W}} -M_0\sum _{i\in \{0,1,\dots ,n,G\}}{{\widetilde{\sigma }}_{ji}\alpha _i{\widetilde{\phi }}_i}, \end{aligned}$$

where ${\widetilde{\psi }}_{ji}$ is the gain of reserve *j* per unit machinery of type *i*, ${\widetilde{\sigma }}_{ji}$ is a stoichiometric coefficient for the synthesis of machinery of type *i*, and ${\widetilde{\sigma }}_{jW}$ is a stoichiometric coefficient for growth. The last coefficient can be further analysed into an assimilatory component, i.e. reserve *j* is used as building block, and a dissimilatory component, i.e. *j* is used as energy source; in general, reserve *j* might be used in both ways and ${\widetilde{\sigma }}_{jW}$ represents the net effect. Growth proceeds in proportion to the quantity of machinery that is dedicated to it:

4 $$\begin{aligned} {\dot{W}}={\widetilde{\psi }}_W M_G, \end{aligned}$$

where ${\widetilde{\psi }}_W$ is a stoichiometric coefficient. The specific growth rate equals $W^{-1}{\dot{W}}$.

Let ${\widehat{\widetilde{\phi _i}}} f_i M_i$ denote the flux of nutrient molecules through assimilatory machinery of type *i*, where ${\widehat{\widetilde{\phi _i}}}$ is a maximum rate and $f_i\in [0,1]$ depends on ambient conditions and possibly also on modulation by cellular factors (Deutscher et al. 2014; Hariharan et al. 2015). Suppose that $E^{(1)}$, $E^{(2)}$, $\dots$ are the elements of interest. These could be any subset of the biogenic elements (C, H, O, N, S, P,...) but in fact, any functional group or carbon skeleton that is not transformed by the metabolism of the organism of interest can be treated as an ‘element.’ For the sake of simplicity, we take the number of elements of interest to be equal to the number of reserves *n*. Nutrient *i* has chemical formula $E_{\nu _{1i}}^{(1)},\;E_{\nu _{2i}}^{(2)}\;E_{\nu _{3i}}^{(3)}\ldots E_{\nu _{ni}}^{(n)}$, where the subscript $\nu _{ki}$ is the number of element *k* in a molecule of nutrient *i*. The chemical composition of the nutrients can be collected in an $n\times n$ matrix ${\mathbf {N}}$ whose *i*th column is $[\nu _{1i},\nu _{2i},\nu _{3i},\ldots ,\nu _{ni}]^T$. Similarly, the chemical composition of the reserves can be represented in an $n\times n$ matrix ${\mathbf {R}}$ whose *j*th column is the formula of reserve *j*. Inasmuch as reserve compounds are chemically distinct for different nutrients, we can assume that the inverse ${\mathbf {R}}^{-1}$ exists. We then have an explicit expression for the stoichiometric coefficient ${\widetilde{\psi }_{ji}}$:

5 $$\begin{aligned} {\widetilde{\psi }_{ji}}=\gamma _{ji}{\widehat{\widetilde{\phi _i}}} f_i, \end{aligned}$$

where $\gamma _{ji}$ denotes the (*j*, *i*)th element of ${\mathbf {R}}^{-1}\cdot {\mathbf {N}}$.

#### Scaling

Choosing suitable parameters as natural units, we may render the equations dimensionless, which can facilitate the analysis of a mathematical model (van den Berg 2011). Adopting ${\widetilde{\phi }}_0^{-1}$ as unit of time, we define scaled variables as follows:

6 $$\begin{aligned} m_i=\frac{M_i {\widetilde{\phi }}_0}{W{\widehat{m}}{\widetilde{\phi }}_i}; \quad x_j=\frac{X_j}{W{\widetilde{\sigma }}_{jW}}. \end{aligned}$$

Here ${\widehat{m}}$ is a scaling parameter for $M_0/W$; its significance will be discussed in Sect. 2.2. Scaled stoichiometric parameters are defined as follows:

7 $$\begin{aligned} \psi _{ji}=\frac{{\widetilde{\psi }}_{ji}{\widetilde{\phi }}_i{\widehat{m}}}{{\widetilde{\sigma }}_{jW}{\widetilde{\phi }}_0^{2}}; \quad \psi _{W}=\frac{{\widetilde{\psi }}_{W}{\widetilde{\phi }}_G{\widehat{m}}}{{\widetilde{\phi }}_0^{2}}; \quad \sigma _{ji}=\frac{{\widetilde{\sigma }}_{ji}{\widetilde{\phi }}_i{\widehat{m}}}{{\widetilde{\sigma }}_{jW}{\widetilde{\phi }}_0}. \end{aligned}$$

On this scaling, the specific growth rate $(W{\widetilde{\phi }}_0)^{-1}{\dot{W}}$ is equal to $\psi _W m_G$; it is convenient to give this quantity its own symbol $\mu$. The biochemical similarity of different types of machinery implies that the relative amounts of reserves expended on their synthesis will be similar as well. This motivates the assumption that for every reserve *j*, we have $\sigma _{ji}=\sigma _j$ for all machineries *i*. The scaled state variables $\{m_0,\dots ,m_G,x_1,\dots , x_n\}$ represent densities: these are intensive variables, as opposed to the original variables $\{M_0,\dots ,M_G,X_1,\dots , X_n\}$, which are extensive (i.e, $\propto W$). After scaling, we have the following dynamics:

8 $$\begin{aligned} \left\{ \begin{array}{lll} {\dot{m}}_i &{}=\alpha _i m_0-\mu m_i &{}\quad \text {for }\ i\in \{0,1,\dots ,n,G\}\\ {\dot{x}}_j &{}=\sum _{i=1}^{n}{\psi _{ji} m_i}-\mu \left( 1+x_j\right) -m_0\sigma _j&{}\quad \text {for }\ j\in \{1,\dots ,n\}. \end{array} \right. \end{aligned}$$

For the sake of simplicity, we shall assume henceforth that $\psi _{ji}=0$ whenever $j\ne i$. This is reasonable when the elemental ratios of the reserves are identical, or nearly identical, to the elemental ratios in the nutrients, since in that case ${\mathbf {R}}\propto {\mathbf {N}}$ and hence ${\mathbf {R}}^{-1}\cdot {\mathbf {N}}$ will be diagonal. Choosing the elements of interest judiciously can also ensure that the matrix $\varvec{\Psi }$ is diagonal. For instance, for *E. coli* growing on glucose and ammonia, only the off-diagonal elements corresponding to hydrogen and oxygen are non-zero; focussing on only carbon and nitrogen, we obtain a $2\times 2$ diagonal matrix.

### Constitutive relationships

To complete the specification of the model, we require expressions for the allocation coefficients $\alpha _0, \ldots , \alpha _G$. One option is to treat these as forcing functions that drive the model. These functions can be observed directly, due to recent advances in ribosome profiling (Ingolia et al. 2009; Li et al. 2014) and enzyme re-profiling (Kramer et al. 2010). Alternatively, the allocation coefficients can be treated as control inputs, to be calculated on the basis of a suitable, evolutionarily relevant optimality criterion (van den Berg et al. 1998). Another option to ‘close’ the equations is to posit outright the dynamics for the reserve densities $x_i$, for instance setting ${\dot{x}}_i=\nu _i\left( f_i-x_i\right)$, where $\nu _i$ is a positive constant (Kooijman 2009) and $f_i$ as in Eq. (5). This approach, which defines the allocation implicitly, while having the advantage of simple dynamics, would seem to require cellular-level stoichiometric parameters to be in fortuitous agreement with the kinetic parameters of the molecules of the regulatory system (van den Berg 1998a). Here, we treat the allocation coefficients as a function of the internal state variables and/or environmental parameters (Parnas and Cohen 1976). In particular, we assume that $m_0,\dots ,m_G,x_1,\dots , x_n$ are mapped to $\alpha _0,\dots ,\alpha _G$ by a suitable ${\mathbb {R}}^{2(n+1)}\mapsto {\mathbb {R}}^{2(n+1)}$ function. Recalling that $\alpha _i$ is the fraction of ribosome time devoted to the production of machinery of type *i*, we propose the following:

9 $$\begin{aligned} \alpha _i=\frac{{\widetilde{r}}_i}{{\widetilde{r}}_0+{\widetilde{r}}_1+\cdots +{\widetilde{r}}_n+{\widetilde{r}}_G}, \end{aligned}$$

where the ${\widetilde{r}}_i$ represent, roughly speaking, the concentrations of translationally active mRNA for the corresponding types of machinery (corrected for relevant molecular properties, such as affinity for the ribosome and mobility within the cytosol, which we tacitly assume can be done via suitable weighting coefficients; synthesis rates in *E. coli* are predominantly under translational, rather than transcriptional control (Li et al. 2014). For the sake of simplicity, ${\widetilde{r}}_0$ is assumed to be constant, corresponding to constitutive expression. We scale the other ${\widetilde{r}_i}$ by this constant:

10 $$\begin{aligned} r_i={{\widetilde{r}}_i}/{{\widetilde{r}}_0}. \end{aligned}$$

For $j=1,\dots , n$, $r_j$ is assumed to be a decreasing function of $x_j$ (we shall take this as a generic sigmoid for the sake of convenience); as the reserve density increases, less of the machinery that feeds it is synthesised. The central mechanism in the present theory resides in a feedback loop connecting reserve densities and allocation of building blocks to machinery; the control logic here is related to that of I-control in control engineering (cf. Jacobs 1993). The building blocks are fed from core metabolism into the synthesis routes; the allotment is achieved effectively by an allocation of ribosome time (cf. the Scott-Hwa-model; Scott et al. 2010, 2014; Scott and Hwa 2011). The $r_j$ can be thought of as corresponding to levels of mRNA for the various types of molecular machinery, although issues such as differences in stability of the mRNA molecule, affinity for ribosomes may distort a direct 1-to-1 correspondence (which can be compensated to some extent by assuming that appropriate correction factors have been assimilated into the scaling).

We assume further that $r_G$ is an increasing function of $m_0$. For the sake of simplicity, we represent it as a piecewise affine function:

11 $$\begin{aligned} r_G [m_0]= \left\{ \begin{array}{l@{\quad }l} 0&{}\text {if }\ m_0\le 1-\epsilon \\ {r_{G, \mathrm {max}}}/{2} + K(m_0 - 1)\;&{}\text {if }\ 1-\epsilon < m_0\le 1+\epsilon \\ r_{G, \mathrm {max}}&{}\text {if }\ m_0 > 1+\epsilon , \end{array} \right. \end{aligned}$$

where *K* is the slope, and $\epsilon = {r_{G, \mathrm {max}}}/{(2K)}$. The midpoint of this function is set at $m_0=1$ (we here exercise our freedom to choose a natural unit for the scaling factor ${\widehat{m}}$ which we identify as the physiological optimum for type-zero machinery; $m_0=1$ follows from this choice). Equation (11) expresses the hypothesis that the safeguarding of core catalytic machinery takes precedence over growth (Bleecken 1988). This relationship is suggested by, and consistent with, Herbert (1961) classic observations on the relationship between RNA content and growth rate (the component $m_0$ corresponding to rRNA). The slope of the relationship observed by Herbert (1961) is inversely proportional to *K*, that is, the larger the value of *K*, the smaller the variation of RNA content with growth rate.

## Consistency with classic models; observability

In this section we investigate the case $n=1$ in more detail, with an emphasis on the continuity of the present approach with the classic empirical laws proposed by Monod (1949) and Droop (1968). In addition, we discuss how the function $r_1$ can be observed by transforming available observational data in a suitable way. This is important since the *r*-functions are the only non-standard (and possibly controversial) constituents of the model, as its remaining assumptions are closely linked to the law of conservation of mass.

### Equilibrium conditions for $n=1$

System (8) takes on the following form for $n=1$:

12 $$\begin{aligned} {\dot{m}}_0= & {} \alpha _0 m_0-\mu m_0;\quad {\dot{m}}_1 =\alpha _1 m_0-\mu m_1;\nonumber \\ {\dot{m}}_G= & {} \alpha _G m_0-\mu m_G;\quad {\dot{x}}_1 =\psi _{1} m_1-\mu \left( 1+x_1\right) -\sigma _1 m_0, \end{aligned}$$

with allocation fractions

$$\begin{aligned} \alpha _0=\frac{1}{1+r_1+r_G};\quad \alpha _1=\frac{r_1}{1+r_1+r_G};\quad \alpha _G=\frac{r_G}{1+r_1+r_G}. \end{aligned}$$

At equilibrium, the rates of change in system (12) equal zero, which can be reduced, via the considerations presented in Sect. 5.1 for general *n*, to the following pair of equilibrium conditions:

13 $$\begin{aligned} \mu&=\psi _W r_G m_0=\left( 1+r_G+r_1\right) ^{-1}\end{aligned}$$

14 $$\begin{aligned} \psi _1 r_1&=\psi _W r_G (1+x_1)+\sigma _1. \end{aligned}$$

Provided that $r_{G,\mathrm {max}}$ is sufficiently large (which is biologically plausible) the state variable $m_0$ can be assumed to lie in the interval $(1-\epsilon ,1+\epsilon )$. We then have the bounds $\psi _1^{\{+\epsilon \}}<\psi _1<\psi _1^{\{-\epsilon \}}$ and $\mu ^{\{-\epsilon \}}<\mu <\mu ^{\{+\epsilon \}}$, where

15 $$\begin{aligned} \psi _1^{\{\pm \epsilon \}}&= \frac{\sigma _1}{r_1(x_1)}+\frac{\psi _W\left( 1+x_1\right) }{2 r_1(x_1)}\left( \sqrt{\frac{4}{\psi _W(1\pm \epsilon )}+\left( 1+r_1(x_1)\right) ^2}-\left( 1+r_1(x_1)\right) \right) \end{aligned}$$

16 $$\begin{aligned} \mu ^{\{\pm \epsilon \}}\!&=\!\frac{1}{2}\left( \sqrt{\psi _W(1\pm \epsilon )\left( 4\!+\!\psi _W(1\pm \epsilon )\left( 1\!+\!r_1(x_1)\right) ^2\right) }\!-\!\psi _W(1\pm \epsilon )\left( 1\!+\!r_1(x_1)\right) \right) \end{aligned}$$

and we have written $r_1(x_1)$ to emphasise that $r_1$ is a function of $x_1$. For small $\epsilon$, these bounds converge and Eqs. (15) and (16) furnish simple expressions for the steady-state relationships between $\psi _1$, $\mu$, and $x_1$; this limit obtains when *K* is sufficiently large.

### Observability of the function $r_1(\cdot )$

Two constitutive functions remain to be specified: the dependence of $\psi _1$ on the ambient concentration of the nutrient, and the function $r_1(\cdot )$. For the former, the Michaelis-Menten hyperbola is a standard choice (van den Berg 2011):

17 $$\begin{aligned} \psi _1={{\widehat{\psi }}_1}\left( {1+K_{\psi ,1}/[\text {N}_1]}\right) ^{-1}, \end{aligned}$$

where ${\widehat{\psi }}_1$ and $K_{\psi ,1}$ are positive parameters and $[\text {N}_1]$ is the ambient nutrient concentration.

**Fig. 2:**
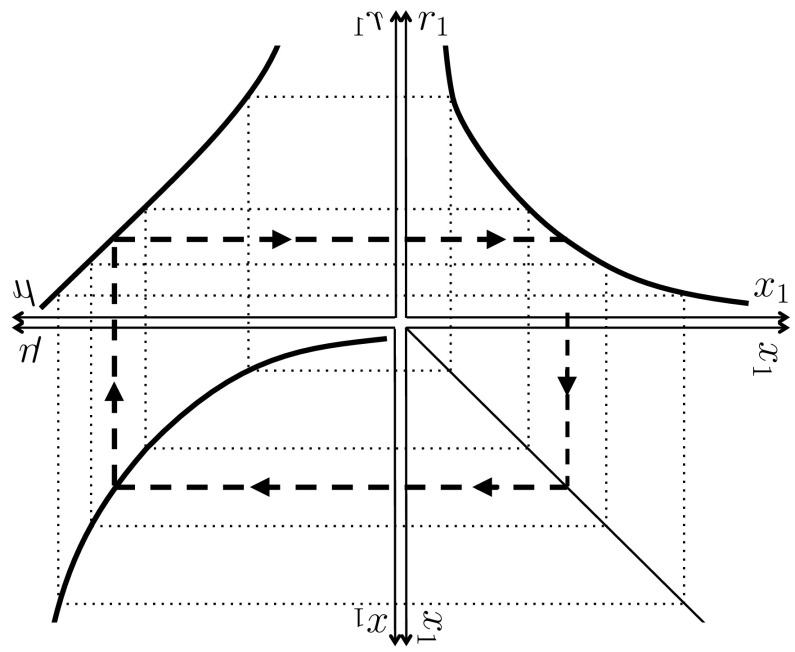
Graphical reconstruction of the function $r_1(\cdot )$. If there are known steady-state relationships between reserve density $x_1$ and specific growth rate $\mu$ (*bottom left*, printed *upside down*), and between $r_1$ and $\mu$ (*top left*, mirror reversed), the function $r_1$ can be plotted in dependence of $x_1$ (*top right*) by chasing set values of $x_1$ around the diagram, as shown by the *dashed arrows*

**Fig. 3:**
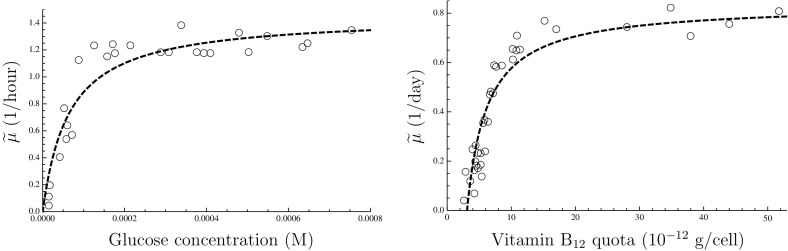
Empirical laws. *Left* steady-state relationship between ambient nutrient concentration and specific growth rate. *Escherichia coli* data from Monod (1949), together with the optimal non-linear least-squares fit of his model, Eq. (20): $K_{\mu ,1}=6.39\times 10^{-5}$ M; ${\widehat{\mu }}=1.45$ h$^{-1}$. *Right* steady-state relationship between cell quota and specific growth rate. *Monochrysis lutheri* data from Droop (1968), together with the optimal non-linear least-squares fit of his model, Eq. (22): $Q_{10}=3.09\times 10^{-12}$ g/cell; ${\widehat{\mu }}=0.835$ day$^{-1}$

The function $r_1(\cdot )$ can be recovered from observational data as shown in Fig. 2, via the parametric dependence of $x_1$ and $r_1$ on $\mu$ at steady state. Under strict homeostasis of type-zero machinery (a condition which we will denote as $m_0\doteq 1$) we have

18 $$\begin{aligned} x_1&=\psi _1 \left( \mu ^{-2}-{\mu }^{-1}-{\psi _W}^{-1}\right) -\left( 1+\sigma _1/\mu \right) \end{aligned}$$

19 $$\begin{aligned} r_1&={\mu }^{-1}-1-{\mu }/{\psi _W} \end{aligned}$$

which means that the construction of Fig. 2 can be carried out if the steady-state relationship between $\psi _1$ and $\mu$ is available. Equation (17) can be used to recover this curve if $\mu$ is known as a function of $[\text {N}_1]$. The latter relationship is the *Monod curve*, an example of which is shown in Fig. 3, which shows data obtained by Monod (1949) along with the hyperbola which he proposed as an empirical law:

20 $$\begin{aligned} {\widetilde{\mu }}={\widehat{\mu }}(1+K_{\mu ,1}/[\text {N}_1])^{-1}, \end{aligned}$$

where ${\widehat{\mu }}$ and $K_{\mu ,1}$ are positive parameters and ${\widetilde{\mu }}$ is the measured specific growth rate in an appropriate SI unit (by scaling, ${\widetilde{\mu }}=\mu {\widetilde{\phi }}_0$). The resemblance to Eq. (17) is obvious, although $K_{\psi ,1}\ne K_{\mu ,1}$ (Button 1991); Monod (1949) pointed out that $K_{\mu ,1}$ can be one or several orders of magnitude smaller than $K_{\psi ,1}$.

Alternatively, the $x_1$-$\mu$ curve may be derived from an empirical law. Reserve density can in some cases be observed directly, when the reserve takes the form of discrete inclusions. However, in other cases, the reserve is composed of molecules that are identical to those occurring as part of the machinery components—for instance, RNA can serve as both functional machinery and reserve, in which case the partitioning between these components is formal but not physical (the individual molecules are not distinguishable as belonging to one or the other). However, it is always possible to observe the total amount per cell, known as the *cell quota*, which can be related to the components via a linear stoichiometric combination:

21 $$\begin{aligned} Q_1=\kappa _W+\kappa _{m,0} m_0+\kappa _{m,1} m_1+\kappa _{m,G} m_G+\kappa _{x,1}x_1, \end{aligned}$$

where the $\kappa _{\star }$ account for the amount of nutrient that is incorporated per unit of the corresponding component $\star$. An empirical law relating $Q_1$ and $\mu$ is known as a *Droop curve*, after Droop (1968) who proposed the following empirical relationship:

22 $$\begin{aligned} {\widetilde{\mu }}={\widehat{\mu }}\left( 1-Q_{10}/Q_1\right) , \end{aligned}$$

where ${\widehat{\mu }}$ and $Q_{10}$ are positive parameters (see Fig. 3). In the special case $m_0\doteq 1$, Eqs. (21) and (22) yield:

23 $$\begin{aligned} x_1=\frac{Q_{10}/\kappa _{x,1}}{1-\mu \widetilde{\phi _0}/{\widehat{\mu }}} -\frac{\kappa _{m,1}}{\kappa _{x,1}\mu } +\mu \frac{\kappa _{m,1}-\kappa _{m,G}}{\kappa _{x,1}\psi _W} -\frac{\kappa _W+\kappa _{m,0}-\kappa _{m,1}}{\kappa _{x,1}} \end{aligned}$$

which furnishes the curve needed for the first transformation in Fig. 2 (bottom left panel), the second transformation being given by Eq. (19), as before.

### Strict reserve homeostasis and the transient Monod model

A case of special interest is that of *strict homeostasis of the reserve* $x_1$. Let

24 $$\begin{aligned} r_1={\widehat{r}}_1\left( 1+\exp \left\{ \vartheta _1\left( x_1-\xi _1\right) \right\} \right) ^{-1}, \end{aligned}$$

where ${\widehat{r}}_1$, $\vartheta _1$ and $\xi _1$ are positive parameters (any generic sigmoid function will do for the purpose at hand). Consider the limit $\vartheta _1\rightarrow \infty$; the function becomes infinitely steep in the neighbourhood of $x_1=\xi _1$, so that $x_1$ remains close to $\xi _1$ over most of the physiological range (excepting perhaps at low growth rates). We shall denote this special case as $x_1\doteq \xi _1$. Combining this with Eq. (17) and $m_0\doteq 1$, we obtain the following relationship between $[\text {N}_1]$ and ${\widetilde{\mu }}$:

25 $$\begin{aligned} \frac{{\widehat{\psi }}_1/\left( 1+\xi _1\right) }{1+K_{\psi ,1}/[\text {N}_1]} = \frac{\sigma _1/\left( 1+\xi _1\right) +{\widetilde{\mu }}/\widetilde{\phi _0}}{\widetilde{\phi _0}/{\widetilde{\mu }}-1-{\widetilde{\mu }}/(\widetilde{\phi _0}\psi _W)}. \end{aligned}$$

This relationship has five free parameters, which is too many to be determined by least-squares fitting from Monod’s data in Fig. 3 alone, but good agreement with the data can be attained (in suitable limits for the parameters, the solution for ${\widetilde{\mu }}$ of Eq. (25) reduces to Monod’s hyperbola).

The ordinary differential equation

26 $$\begin{aligned} {\dot{W}}= W {\widehat{\mu }}\left( 1+K_{\mu ,1}/[\text {N}_1]\right) ^{-1} \end{aligned}$$

is often referred to as the “Monod model” (e.g., Dawes 1989; van Gemerden 1993; de Wit et al. 1995), where $[\text {N}_1]$ is treated as an autonomous function of time or coupled to *W* via a suitable ecological model (for instance, if the culture is growing under batch conditions, $[\text {N}_1]$ will decrease as *W* increases). Equation (26) is more accurately called the *transient Monod* model to indicate that application to transient conditions ventures beyond the steady-state originally considered by Monod. The transient Monod model has just one component (*W* is its only state variable); when it occurs as part of an ecological model, stoichiometric consistency requires that $x_1\doteq \xi _1$, so the assumption of strict reserve homeostasis must be imputed to such studies even when the authors do not explicitly commit to this.

The behaviour of the present model under transient conditions differs from the transient Monod model, even under the assumption that the function $r_1(x_1)$ has a steep slope ($\vartheta _1\rightarrow \infty$). Following a change in environmental conditions, for instance a step change in $[\text {N}_1]$, $x_1$ deviates from $\xi _1$ which triggers a re-allocation of building blocks to the various types of machinery. As $[\text {N}_1]$ is held constant at its new value, the *m*-type state variables relax at a time scale $\sim \mu ^{-1}$; while $x_1$ relaxes back to $\xi _1$.

However, the transient Monod model and the present model can be treated as equivalent if the changes in $[\text {N}_1]$ (and hence $\psi _1$) occur smoothly and sufficiently slowly. In this ‘adiabatic’ case, the internal dynamics is sufficiently rapid that its state variables can be coupled quasi-statically to $[\text {N}_1]$, or, equivalently, to $\psi _1$ (cf. Sect. 4.1).

## Simulations

The dynamics, system (8), can be studied in qualitative terms by means of numerical solution of the ordinary differential equations. In this section we note several aspects of the model’s dynamic behaviour which could be measured, in principle, in the real-life system.

### The case $n=1$

**Fig. 4:**
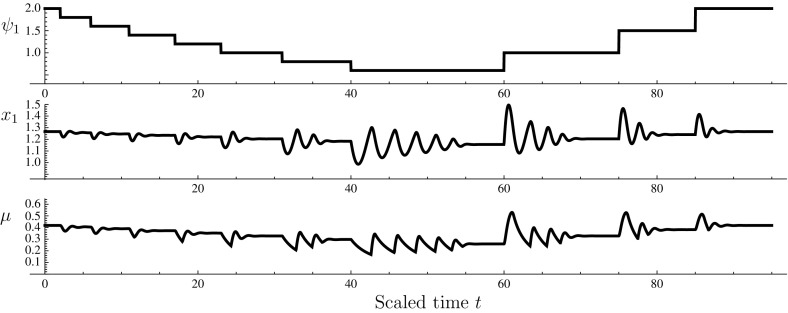
Numerical solution of system (12). The function $r_G$ was as in Eq. (11) with $K=10^4$ and $r_{G,\mathrm {max}}=5$; $r_1=15/\left( 1+\exp \{10(x_1-1)\}\right)$; $\psi _W=1$; and $\sigma _1=1$. *Top* imposed time course of $\psi _1$. *Middle* time course of scaled reserve density $x_1$. *Bottom* time course of the specific growth rate $\mu$

The response of the model to stepwise increases and decreases of $\psi _1$ is shown in Fig. 4. It can be seen that the downward steps in $\psi _1$, representing a decrease in ambient nutrient availability, lead to downward deflections in the reserve $x_1$ which governs the dynamic allocation between nutrient uptake machinery and proliferative (growth) machinery. The adjustment is rapid and stabilises, although oscillations become more vigorous and long-lasting at lower values of $\psi _1$; intuitively this can be understood since the actual balance between $m_1$ and $m_G$ (i.e., the proteome-level profile) relaxes toward the balance dictated by $\alpha _1$ and $\alpha _G$ with a response time of order $\mu ^{-1}$. Thus, even if the change in expression of different kinds of machinery is rapid, the actual balance reacts more sluggishly at low $\mu$. Upward step changes in $\psi _1$ induce upward deflections of $x_1$, which again steer the dynamic re-allocation process.

**Fig. 5:**
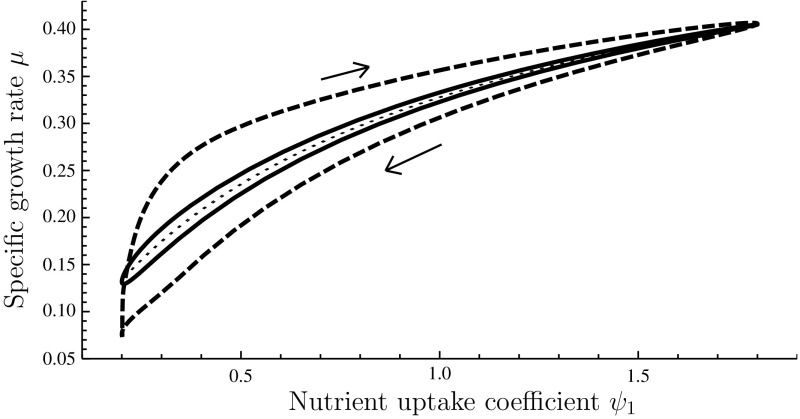
Numerical solution of system (12); stationary cycle under a sinusoidal variation of $\psi _1$. The *dashed curve* obtains for a cycle duration of 60 units of scaled time; the *solid curve* for a cycle duration of 300 units. Also shown is the ‘adiabatic’ limit (*dotted line*) which obtains for an infinitely slow cycle

If a sinusoidal variation in $\psi _1$ is imposed, the system settles on a stationary cycle. Parametric plots of $\psi _1(t)$ versus $\mu (t)$ over this stationary cycle are shown in Fig. 5 for selected values of the period of the cycle. A hysteresis effect is in evidence, which corresponds, loosely speaking, to the proteome-level re-profiling dynamics lagging behind the prevailing value of $\psi _1$. The hysteresis loop widens as the period of the environmental oscillation shortens. As this duration goes to infinity, the loop tightens up against a curve which corresponds to the ‘adiabatic’ regime under which the transient Monod model is valid: provided that environmental changes are sufficiently slow, $\mu$ can be treated as a function of the environmental conditions.

**Fig. 6:**
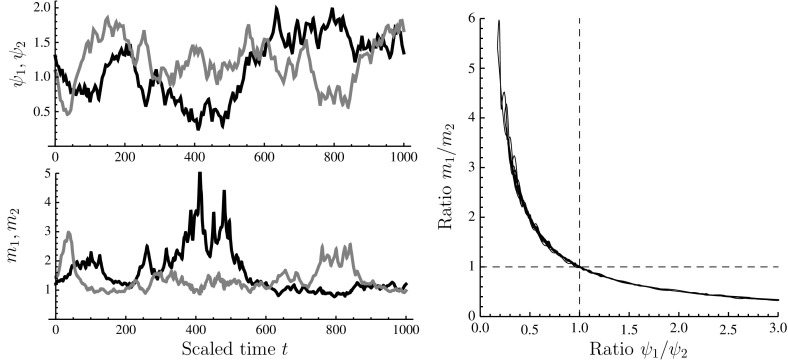
Numerical solution of system (8) with $n=2$. The function $r_G$ was as in Eq. (11) with $K=10^4$ and $r_{G,\mathrm {max}}=5$; $r_i=15/\left( 1+\exp \{10(x_i-1)\}\right)$ and $\sigma _i=1$ for $i=1,2$; and $\psi _W=1$. *Left*, *top* imposed time course of $\psi _1$ (*black line*) and $\psi _2$ (*grey line*). *Left*, *bottom* time course of scaled uptake machinery for nutrient 1 ($m_1$; *black line*) and nutrient 2 ($m_2$; *grey line*). Right: $\psi _1/\psi _2$ versus $m_1/m_2$, showing compensatory shifts in expression of nutrient uptake machinery

### The case $n=2$

For two or more reserve components, the assumption of monotonically decreasing $r_i$-functions leads to a re-balancing effect, whereby stoichiometric imbalances between nutrient availabilities are offset, or at least partially offset, by counteracting changes in the allocation fractions to the corresponding types of uptake machinery.

This ‘counter-skewing’ effect is illustrated in Fig. 6, in which the model was simulated for $n=2$ with uncorrelated white noise in the $\psi _1$ and $\psi _2$ time-courses. It can be seen that $m_i$ tends to increase when $\psi _i$ is relatively low, and vice versa. Indeed, when the ratio $\psi _1/\psi _2$ is plotted against $m_1/m_2$ over the time course of the simulation run, a perfect hyperbola is obtained.

The model achieves this behaviour by having each reserve feeding back on the expression of the machinery feeding that particular reserve; the balancing in allocation happens at the level of Eq. (9), which represents the effect of ribosomes distributing themselves pro rata over the mRNA species, as we would expect based on the random encounter processes that underlie molecular kinetics. This shows that it is possible in principle to achieve reserve homeostasis without the need for signals arising from multiple reserves to converge on the upstream activation sequence of any one of the genes for uptake machinery.

**Fig. 7:**
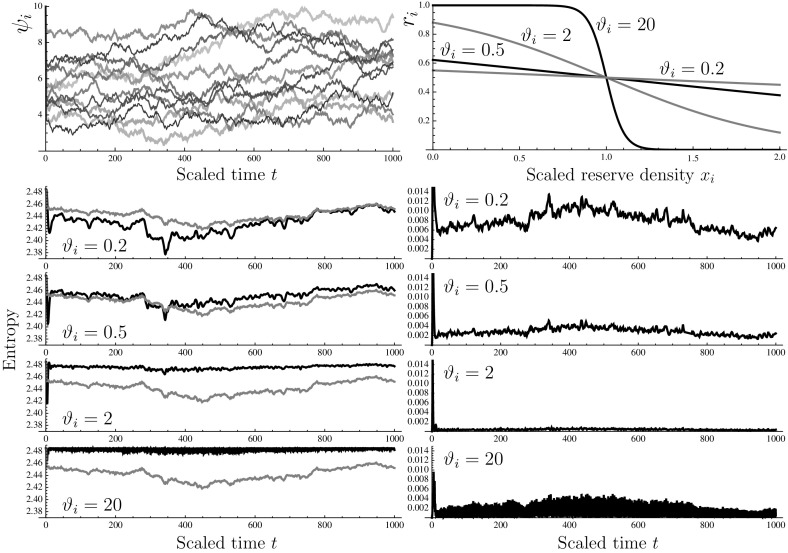
Numerical solution of system (8) with $n=12$, with initial condition corresponding to optimal environment, i.e., $\psi _i\equiv {\widehat{\psi }}_i$ for all *i*. *Top left* uncorrelated white noise functions for $\psi _i$ ($i=1,\dots ,12$) used in all simulations. *Top right* sigmoid functions used for $r_i$ in the simulations; all reserves use the same function in any given run, but the steepness parameter $\vartheta _i$ was varied as shown. *Bottom left* time course of the reserve entropy (*solid line*) for various values of $\vartheta _i$; the input entropy of the $\psi _i$ is shown for reference as a *grey line*. Reserve entropy was defined as $\sum _{i=1}^{n}{(x_i/x_T)}\ln \left\{ x_T/x_i\right\}$ with $x_T=\sum _{i=1}^{n}{x_i}$; Ambient entropy was defined as $\sum _{i=1}^{n}{(\psi _i/\psi _T)}\ln \left\{ \psi _T/\psi _i\right\}$ with $\psi _T=\sum _{i=1}^{n}{\psi _i}$. *Bottom right* time course of the relative entropy for various values of $\vartheta _i$; relative entropy was defined as $\sum _{i=1}^{n}{(m_T/m_i})\ln \left\{ m_T\psi _T/(m_i\psi _i)\right\}$ with $m_T=\sum _{i=1}^{n}{m_i}$

### Multiple reserve components

The qualitative behaviours noted in the foregoing sections are also present at $n\ge 3$. By way of example, the response of a model with $n=12$ is shown in Fig. 7. Again, the ambient medium presents uncorrelated noise. The response to this environmental input is represented for four different steepness values [$\vartheta _i$, see Eq. (24)] of the $r_i$-functions. When this value is low, the allocation to the uptake machineries is hardly adjusted. As a result, the entropy of the reserves follows that of the environment; in other words, the fluctuations in the environment are reflected in fluctuations in reserve status (in the simulation shown for $\vartheta _i=0.2$, the reserve entropy temporarily undershoots the ambient entropy; this is a transient effect due to the initial state of the system).

As $\vartheta _i$ increases, the reserve entropy tends more and more toward the maximum value ($\ln 12\approx 2.485$) at all times, reflecting that strict homeostasis of reserves is achieved. In fact, at the highest value studied ($\vartheta _i=20$) the reserve entropy signal is rather restless, with rapid downward spikes that arise as a consequence of the high reactivity of the feedback loop, which tends to induce rapid oscillations.

To visualise the ‘counter-skewing’ effect, the relative entropy of $\{m_i^{-1}\}_{i=1}^{n}$ with respect to $\{\psi _i\}_{i=1}^{n}$ has been plotted as a function of time. This relative entropy decreases with increasing $\vartheta _i$, indicating that the machinery allocation becomes better adapted to the environmental fluctuations. Again, the trace for $\vartheta _i=20$ appears more agitated than that for $\vartheta _i=2$, due to the rapid oscillations concomitant with high reactivity.

## Dynamics of the model for general *n*

We investigate the existence, uniqueness, and stability of equilibria of system (8). Setting the rates of change equal to zero yields the equilibrium conditions:

27 $$\begin{aligned} \alpha _i m_0-\mu m_i&= 0\quad \text {for }\ i\in \{0,1,\dots ,n,G\}, \end{aligned}$$

28 $$\begin{aligned} \psi _{j} m_j-\mu \left( 1+x_j\right) -m_0\sigma _j&= 0 \quad \text {for }\ j\in \{1,\dots ,n\}. \end{aligned}$$

We shall assume throughout that $\varvec{\Psi }$ is a diagonal matrix, and we are primarily interested in the case where *K* is large (corresponding to strict homeostasis of $m_0$).

### Existence and uniqueness of the equilibrium point

Specifying Eq. (27) for $i=0$, we obtain $\mu =\alpha _0$ (since $m_0>0$ for a biologically relevant equilibrium). Thus Eq. (27) can be written as $m_i=(\alpha _i/\alpha _0)m_0$. With Eq. (9), this becomes $m_i=(r_i/r_0)m_0$ or $m_i=r_i m_0$ since $r_0\equiv 1$ by scaling. In particular, $m_G= r_G m_0$ and hence $\mu =\psi _W r_G m_0$ (since $\mu =\psi _W m_G$ by definition). With these identities, Eq. (28) becomes:

$$\begin{aligned} \left( \psi _{j} r_j-\psi _W r_G \left( 1+x_j\right) - \sigma _j \right) m_0= 0, \end{aligned}$$

which means that either $m_0=0$, which is not biologically relevant, or

29 $$\begin{aligned} \psi _W r_G\left( 1+x_j\right) +\sigma _j = \psi _j r_j . \end{aligned}$$

Let us first consider the problem of solving this for $x_j$ given a fixed value of $r_G\in [0,r_{G,\mathrm {max}}]$. The left-hand side of Eq. (29) is a strictly increasing function of $x_j$ whereas its right-hand side is a strictly decreasing function of $x_j$ (by the assumed properties of the $r_j$ as functions of the $x_j$). The graphs of these two functions intersect in at most one point. This point will exist if the graph of the left-hand side of Eq. (29) lies below that of the right-hand side at $x_j=0$. For this it suffices that

30 $$\begin{aligned} r_{j,\mathrm {max}}\ge \left( \psi _W r_{G,\mathrm {max}}+\sigma _j\right) {/}\psi _j, \end{aligned}$$

where $r_{j,\mathrm {max}}$ denotes the value of $r_j$ at $x_j=0$. The physiological interpretation suggests that $r_{j,\mathrm {max}}<+\infty$, so that condition (30) can only be satisfied if $\psi _j>0$ for $j=1,\dots ,n$. Thus, if condition (30) is satisfied, Eq. (29) will have a unique solution $x_j^\star \ge 0$ for all reserves *j*, for the given value of $r_G$. This solution $x_j^\star$ can be treated as a function of $r_G$ as defined by Eq. (29); this function is strictly decreasing. Since the $r_j$ are strictly decreasing in their respective $x_j$, it follows that $\sum {r}\equiv \sum _{j\in {0,1,\dots ,n,G}}{r_j}$ is an increasing function of $r_G$. At equilibrium $\mu =\alpha _0$ and $\alpha _0=r_0/\sum {r}=1/\sum {r}$, whence $\mu =1{/}\sum {r}$ which is a decreasing function of $r_G$, or equivalently, a decreasing function of $m_0$ (since $r_G$ is an increasing function of $m_0$). In addition, $\mu =\psi _W r_G m_0$, which is an increasing function of $m_0$. Again we consider the point of intersection between the graphs of these two functions. Repeating a similar argument, we find that $1/\sum {r}>0$ and $\psi _W r_G m_0=0$ at $m_0=1-\epsilon$, and that therefore it is sufficient if

31 $$\begin{aligned} \left( 1+r_{G,\mathrm {max}}+\sum _{j=1}^{n}{r_{j}^\star (r_{G, \mathrm {max}})}\right) \psi _W r_{G,\mathrm {max}}\ge (1+\epsilon )^{-1} \end{aligned}$$

for a unique intersection point to exist with $1-\epsilon \le m_0\le 1+\epsilon$. We concluded earlier that, at equilibrium $m_G=r_G m_0$ and $m_j=r_j m_0$ for $j=1,\dots ,n$; therefore these are fixed whenever the $x_j$ together with $m_0$ are fixed. Conditions (30) and (31) suffice; they could be weakened but even in the form stated, they are not at all stringent from a biological point of view: it is enough that the $r_j$-functions are sufficiently large for small values of their argument. We henceforth assume that these conditions are met.

### Linear stability analysis

We investigate the stability by linearising the system about its equilibrium and verifying the stability of the characteristic polynomial associated with the linearised system. The system matrix of the linearised system is the Jacobian matrix:

$$\begin{aligned}&J (m_0, m_1\ldots m_n, m_G, x_1\ldots x_n)\\&\quad =\begin{pmatrix} \frac{\partial f_1}{\partial m_0} &{}\quad \frac{\partial f_1}{\partial m_1} &{}\quad \dots &{}\quad \frac{\partial f_1}{\partial m_n} &{}\quad \frac{\partial f_1}{\partial m_G} &{}\quad \frac{\partial f_1}{\partial x_1} &{}\quad \dots &{}\quad \frac{\partial f_1}{\partial x_n}\\ \frac{\partial f_2}{\partial m_0} &{}\quad \frac{\partial f_2}{\partial m_1} &{}\quad \dots &{}\quad \frac{\partial f_2}{\partial m_n} &{}\quad \frac{\partial f_2}{\partial m_G} &{}\quad \frac{\partial f_2}{\partial x_1} &{}\quad \dots &{}\quad \frac{\partial f_2}{\partial x_n}\\ \vdots &{}\quad \vdots &{}\quad \vdots &{}\quad \vdots &{}\quad \vdots &{}\quad \vdots &{}\quad \vdots &{}\quad \vdots \\ \frac{\partial f_{2n+2}}{\partial m_0} &{}\quad \frac{\partial f_{2n+2}}{\partial m_1} &{}\quad \dots &{}\quad \frac{\partial f_{2n+2}}{\partial m_n} &{}\quad \frac{\partial f_{2n+2}}{\partial m_G} &{}\quad \frac{\partial f_{2n+2}}{\partial x_1} &{}\quad \dots &{}\quad \frac{\partial f_{2n+2}}{\partial x_n} \end{pmatrix} , \end{aligned}$$

where $f_1, f_2 \ldots f_{2n+2}$ denote the right-hand sides of system (8). Our strategy is to investigate the signs of the coefficients in the limit $K\rightarrow \infty$ [the parameter *K* represents the slope of the increasing part of the piecewise function $r_G$ in Eq. (11)]. In real-life systems, $K<+\infty$, as attested by the non-zero slope of cellular RNA content ($\sim m_0$) as a function of $\mu$ at steady state (Herbert 1961). In other words, the limit $K\rightarrow \infty$ represents an idealised case of strict homeostasis of $m_0$. While our limiting result establishes that stability is ensured if *K* is sufficiently large, numerical solutions of the dynamic equations for finite *K* indicate that, in fact, the system always converges to its equilibrium point, but, as *K* decreased, with oscillations of increasing amplitude and ring-down time. To establish the result in the limit $K\rightarrow \infty$, we exploit a theorem by Strelitz (1977) on the stability of monic polynomials.

#### Signs of the coefficients of the characteristic equation

The characteristic equation is a polynomial of order $2(n+1)$:

32 $$\begin{aligned} \lambda ^{2n+2} + c_1\lambda ^{2n+1} + c_2\lambda ^{2n} + \dots + c_{2n+1}\lambda + c_{2n+2} = 0. \end{aligned}$$

The coefficients $c_k$ can be written as $c_k=(-1)^k S_k$, where $S_k$ is the sum of the principal minors $M_k$ (Mishina and Proskuryakov 1962). These $M_k$ are symmetric with respect to the main diagonal of the Jacobian matrix. The coefficients $c_k$ are first-order polynomials in *K*. For sufficiently large *K*, it is the term in $c_k$ that is proportional to *K* that governs the sign; let ${\widetilde{c}_k}$ denote this term.

It will prove useful to partition the Jacobian matrix as follows:

33 $$\begin{aligned} J=\left[ \begin{array} {c|c|c|c} J^{\mathrm {I}}&J^{\mathrm{II}}&J^{\mathrm {III}}&J^{\mathrm {IV}} \end{array} \right] . \end{aligned}$$

The submatrices are evaluated at the equilibrium point in the limit $m_0\rightarrow 1$, that is, $\epsilon \rightarrow 0$ [which obtains as $K\rightarrow \infty$, cf. Eq. (11)]:

$$\begin{aligned} J^{\mathrm {I}}= & {} \begin{pmatrix} -\psi _W^2 Kr_G^2 \\ \psi _W r_1 r_G - \psi _W^2 r_1 Kr_G^2 \\ \vdots \\ \psi _W r_n r_G - \psi _W^2 r_n Kr_G^2 \\ \psi _W Kr_G + \psi _Wr_G^2-\psi _W^2 Kr_G^3 \\ -\sigma _1 \\ \vdots \\ -\sigma _n \end{pmatrix} ,\\ J^{\mathrm {II}}= & {} \begin{pmatrix} 0 &{}\quad \dots &{}\quad 0 \\ -\psi _Wr_G &{}\quad \dots &{}\quad 0 \\ \vdots &{}\quad \vdots &{}\quad \vdots \\ 0 &{}\quad \dots &{}\quad -\psi _Wr_G \\ 0 &{}\quad \dots &{}\quad 0 \\ \psi _{1} &{}\quad \dots &{}\quad 0 \\ \vdots &{}\quad \vdots &{}\quad \vdots \\ 0 &{}\quad \dots &{}\quad \psi _{n} \end{pmatrix} ,\; \quad J^{\mathrm {III}} = \begin{pmatrix} -\psi _W \\ -\psi _W r_1 \\ \vdots \\ -\psi _W r_n \\ -2\psi _Wr_G \\ \frac{\sigma _1-\psi _1 r_1}{r_G} \\ \vdots \\ \frac{\sigma _n-\psi _n r_n}{r_G} \end{pmatrix} ,\\ J^{\mathrm {IV}}= & {} \begin{pmatrix} -\psi _W^2 r_1'r_G^2 &{}\quad \dots &{}\quad -\psi _W^2 r_n'r_G^2\\ \psi _W r_1'r_G - \psi _W^2 r_1 r_1'r_G^2 &{}\quad \dots &{}\quad -\psi _W^2r_1 r_n'r_G^2 \\ \vdots &{}\quad \vdots &{}\quad \vdots \\ -\psi _W^2r_1' r_n r_G^2 &{}\quad \dots &{}\quad \psi _W r_n'r_G - \psi _W^2 r_n r_n'r_G^2 \\ -\psi _W^2r_G^3 r_1' &{}\quad \dots &{}\quad -\psi _W^2r_G^3 r_n'\\ -\psi _Wr_G &{}\quad \dots &{}\quad 0\\ \vdots &{}\quad \vdots &{}\quad \vdots \\ 0 &{}\quad \dots &{}\quad -\psi _Wr_G \end{pmatrix} . \end{aligned}$$

##### Lemma 1

Only the minors that contain a diagonal element from $J^{\mathrm {I}}$ contribute terms that are proportional to *K*; in particular,

34 $$\begin{aligned} {\widetilde{c}_k}= & {} C_{2n+1}^{k-1}K\psi _W^{k+1}r_G^{k+1} + C_{2n}^{k-2}K\psi _W^{k}r_G^{k-1}\nonumber \\&+\sum _{\ell =1}^{n}C_{2n-(2\ell -1)}^{k-(2\ell +1)}K\psi _W^{k+1-\ell }r_G^{k-\ell }(-1)^{\ell }\nonumber \\&\times \sum _{{\mathbb {S}}\in {\mathscr {P}}_{\ell }\left( \{1,2\ldots ,n\}\right) }\prod _{m\in {\mathbb {S}}}r_{m}'\left( r_G\prod _{m\in {\mathbb {S}}}\psi _{m}+\sum _{m\in {\mathbb {S}}}(\psi _m r_m -\sigma _m)\prod _{j\in {\mathbb {S}}\setminus m}\psi _j\right) \nonumber \\&+\sum _{\ell =1}^{n}C_{2n-2\ell }^{k-(2\ell +2)}K\psi _W^{k-\ell }r_G^{k-\ell -1}(-1)^{\ell }\sum _{{\mathbb {S}}\in {\mathscr {P}}_{\ell }\left( \{1,2\ldots ,n\}\right) }\prod _{m\in {\mathbb {S}}}\psi _{m}r_{m}', \end{aligned}$$

where ${\mathscr {P}}_{\ell }\left( \{1,2\ldots ,n\}\right)$ is the set of all subsets of the set $\{1,2\ldots ,n\}$ with cardinality $\ell$.

For example, for $n=2$ and $k=6$ we have:

35 $$\begin{aligned} {\widetilde{c}_6}= & {} K\psi _W^7r_G^7 + K\psi _W^6r_G^5 \nonumber \\&- K\psi _W^6r_G^5r_1'\Big (\psi _1r_G + (\psi _1r_1 - \sigma _1)\Big ) - K\psi _W^6r_G^5r_2'\Big (\psi _2r_G + (\psi _2r_2 - \sigma _2)\Big ) \nonumber \\&+ K\psi _W^5r_G^4r_1'r_2'\Big (\psi _1\psi _2r_G + \psi _1(\psi _2r_2-\sigma _2)+\psi _2(\psi _1r_1-\sigma _1)\Big ) \nonumber \\&- K\psi _W^5r_G^4\psi _1r_1' - K\psi _W^5r_G^4\psi _2r_2' + K\psi _W^4r_G^3\psi _1\psi _2r_1'r_2'. \end{aligned}$$

##### Proof

Only the minors that contain a diagonal element from the first column contribute terms proportional to *K*, because the minors that do not contain elements from $J^{\mathrm {I}}$ do not contain any term $\propto K$. There are $2^n - 1$ non-trivial subsets of the set of size *n*. To obtain all minors that contribute terms $\propto K$ we have to inspect $2^3 - 1 = 7$ types of minor containing a diagonal element taken from $J^{\mathrm {I}}$, as the ways in which such minors can be composed depends on the number of non-trivial subsets of the set of size 3. One of these types only occurs for $k=2$ (minors based on $J^{\mathrm {I}}$ and $J^{\mathrm {III}}$) and can be subsumed under type iii. This leaves six types to be distinguished; they are defined as being composed of the following, in addition to the element contributed by the column matrix $J^{\mathrm {I}}$: (i) $k-1$ diagonal elements from $J^{\mathrm {II}}$; (ii) $k-1$ diagonal elements from $J^{\mathrm {IV}}$; (iii) $k-2$ diagonal elements from $J^{\mathrm {II}}$ and one from $J^{\mathrm {III}}$; (iv) $k-2$ diagonal elements from $J^{\mathrm {IV}}$ and one from $J^{\mathrm {III}}$; (v) diagonal elements from $J^{\mathrm{II}}$ and $J^{\mathrm {IV}}$ such that their total number is $k-1$; (vi) diagonal elements from $J^{\mathrm{II}}$ and $J^{\mathrm {IV}}$ such that their total number is $k-2$, in addition to an element from $J^{\mathrm {III}}$.

We consider these types in terms and collect the terms proportional to *K*. For the sake of clarity, expressions such as $(-1)^{k-2},\;(-1)^{k-4}$ etc. will be written as $(-1)^k$ and likewise $(-1)^{k-1},\;(-1)^{k-3}$ etc. will be written as $(-1)^{k+1}$. The binomial coefficient $$\left( {\begin{array}{c}n\\ k\end{array}}\right) = {n!}({k! (n-k)!})^{-1}$$ will be denoted as $C_{n}^{k}$. We will make use of the following:

36 $$\begin{aligned} \sum _{i=0}^{k}C_{n}^i C_{n}^{k-i} = C_{2n}^{k}\quad \text {and}\quad C_{n}^{k} + C_{n}^{k-1} = C_{n+1}^{k}. \end{aligned}$$

Minors of type i have the following form:

$$\begin{aligned} \begin{vmatrix} -\psi _W^2 Kr_G^2&\quad 0&\quad \dots&\quad 0 \\ \psi _W r_1r_G - \psi _W^2 r_1 Kr_G^2&\quad -\psi _Wr_G&\quad \dots&\quad 0 \\ \vdots&\quad \vdots&\quad \vdots&\quad \vdots \\ \psi _W r_{k-1} r_G - \psi _W^2 r_{k-1} Kr_G^2&\quad 0&\quad \dots&\quad -\psi _Wr_G \end{vmatrix} = (-1)^k K\psi _W^{k+1}r_G^{k+1}. \end{aligned}$$

We have $C_n^{k-1}$ such minors, because the first column $J^{\mathrm {I}}$ is fixed and we are choosing $k-1$ diagonal elements from a total of *n* elements in block $J^{\mathrm {II}}$. Thus minors of this type contribute $(-1)^k C_n^{k-1} K\psi _W^{k+1}r_G^{k+1}$ to the right-hand side of Eq. (34).

Minors of type ii have the following form:

$$\begin{aligned} \begin{vmatrix} -\psi _W^2 Kr_G^2&\quad -\psi _W^2 r_1'r_G^2&\quad \dots&\quad -\psi _W^2 r_{k-1}'r_G^2\\ -\sigma _1&\quad - \psi _W r_G&\quad \dots&\quad 0 \\ \vdots&\quad \vdots&\quad \vdots&\quad \vdots \\ -\sigma _{k-1}&\quad 0&\quad \dots&\quad - \psi _W r_G \\ \end{vmatrix} = (-1)^k K\psi _W^{k+1}r_G^{k+1} +\cdots \end{aligned}$$

where the dots (here and in what follows) correspond to terms that do not contain *K*. We have $C_n^{k-1}$ such minors, giving a contribution $(-1)^k C_n^{k-1} K\psi _W^{k+1}r_G^{k+1}$ to Eq. (34).

Minors of type iii have the following form:

$$\begin{aligned}&\begin{vmatrix} -\psi _W^2 Kr_G^2&\quad 0&\quad \dots&\quad 0&\quad -\psi _W \\ \psi _W r_1r_G - \psi _W^2 r_1 Kr_G^2&\quad -\psi _Wr_G&\quad \dots&\quad 0&\quad -\psi _W r_1 \\ \vdots&\quad \vdots&\quad \vdots&\quad \vdots&\quad \vdots \\ \psi _W r_{k-2} r_G - \psi _W^2 r_{k-2} Kr_G^2&\quad 0&\quad \dots&\quad -\psi _Wr_G&\quad -\psi _W r_{k-2}\\ \psi _W Kr_G +\psi _Wr_G^2-\psi _W^2 Kr_G^3&\quad 0&\quad \dots&\quad 0&\quad -2\psi _W r_G\\ \end{vmatrix} \\&\quad = (-1)^{k} K\psi _W^{k+1}r_G^{k+1} + (-1)^{k} K\psi _W^{k}r_G^{k-1} + \cdots . \end{aligned}$$

We have $C_n^{k-2}$ such minors and the contribution is therefore $(-1)^{k} C_n^{k-2} \left( K\psi _W^{k+1}r_G^{k+1} + K\psi _W^{k}r_G^{k-1}\right)$.

Minors of type iv have the following form:

$$\begin{aligned} \begin{vmatrix} -\psi _W^2 Kr_G^2&\quad -\psi _W&\quad -\psi _W^2 r_1'r_G^2&\quad \dots&\quad -\psi _W^2 r_i'r_G^2&\quad \dots&\quad -\psi _W^2 r_{k-2}'r_G^2\\ \psi _W Kr_G +\psi _Wr_G^2-\psi _W^2 Kr_G^3&\quad -2\psi _W r_G&\quad -\psi _W^2 r_1'r_G^3&\quad \dots&\quad -\psi _W^2 r_i'r_G^3&\quad \dots&\quad -\psi _W^2 r_{k-2}'r_G^3 \\ -\sigma _1&\quad ({\sigma _1-\psi _1 r_1})/{r_G}&\quad -\psi _W r_G&\quad \dots&\quad 0&\quad \dots&\quad 0\\ \vdots&\quad \vdots&\quad \vdots&\quad \vdots&\quad \vdots&\quad \vdots&\quad \vdots \\ -\sigma _i&\quad ({\sigma _i-\psi _i r_i})/{r_G}&\quad 0&\quad \dots&\quad -\psi _W r_G&\quad \dots&\quad 0\\ \vdots&\quad \vdots&\quad \vdots&\quad \vdots&\quad \vdots&\quad \vdots&\quad \vdots \\ -\sigma _{k-2}&\quad ({\sigma _{k-2}-\psi _{k-2} r_{k-2}})/{r_G}&\quad 0&\quad \dots&\quad 0&\quad \dots&\quad -\psi _W r_G\\\end{vmatrix}. \end{aligned}$$

These contribute the same term as minors of type iii, i.e. $(-1)^{k} C_n^{k-2} \left( K\psi _W^{k+1}r_G^{k+1} + K\psi _W^{k}r_G^{k-1}\right)$. In addition, minors based on the diagonal element of column *i* in block $J^{\mathrm {IV}}$ contribute $(-1)^{k}r_i'(\sigma _i-r_i\psi _i)K\psi _W^k r_G^{k-1}$. For each *i*, there are $C_{n-1}^{k-3}$ ways of making up the remaining $k-3$ elements (which are chosen from a total $n-1$ in block $J^{\mathrm {IV}}$). This yields a total contribution of $(-1)^{k}C_{n-1}^{k-3}\sum _{i\in {\mathscr {P}}_{1}\left( \{1,2\ldots ,n\}\right) }r_i'(\sigma _i-r_i\psi _i)K\psi _W^k r_G^{k-1}$.

Minors of type v have the following form:

$$\begin{aligned} \begin{vmatrix} -\psi _W^2 Kr_G^2&\quad 0&\quad \dots&\quad 0&\quad -\psi _W^2 r_1'r_G^2&\quad \dots&\quad -\psi _W^2 r_{k-1-i}'r_G^2\\ \psi _W r_1r_G - \psi _W^2 r_1 Kr_G^2&\quad -\psi _W r_G&\quad \dots&\quad 0&\quad \psi _W r_1'r_G - \psi _W^2 r_1r_1'r_G^2&\quad \dots&\quad -\psi _W^2r_1r_{k-1-i}'r_G^2 \\ \vdots&\quad \vdots&\quad \vdots&\quad \vdots&\quad \vdots&\quad \vdots&\quad \vdots \\ \psi _W r_{i} r_G - \psi _W^2 r_{i} Kr_G^2&\quad 0&\quad \dots&\quad -\psi _W r_G&\quad - \psi _W^2 r_{i}r_1'r_G^2&\quad \dots&\quad \psi _W^2 r_{i}r_{k-1-i}' r_G^2 \\ -\sigma _1&\quad \psi _{1}&\quad \dots&\quad 0&\quad -\psi _W r_G&\quad \dots&\quad 0\\ \vdots&\quad \vdots&\quad \vdots&\quad \vdots&\quad \vdots&\quad \vdots&\quad \vdots \\ -\sigma _{k-1-i}&\quad 0&\quad \dots&\quad 0&\quad 0&\quad \dots&\quad -\psi _W r_G\\ \end{vmatrix}. \end{aligned}$$

Each minor contributes a term $(-1)^k K\psi _W^{k+1}r_G^{k+1}$. Since we are choosing $k-1$ diagonal elements from blocks $J^{\mathrm {II}}$ and $J^{\mathrm {IV}}$, the multiplicity is $\sum _{i=1}^{k-2}C_n^i C_n^{k-1-i}$, giving a total $(-1)^k\sum _{i=1}^{k-2}C_n^i C_n^{k-1-i} K\psi _W^{k+1}r_G^{k+1}$. Furthermore, each minor containing diagonal elements from column *i* in $J^{\mathrm {II}}$ and column *i* in $J^{\mathrm {IV}}$, contributes $(-1)^{k+1}\psi _i r_i' K\psi _W^k r_G^k$. There are $\sum _{i=0}^{k-3}C_{n-1}^i C_{n-1}^{k-3-i}$ such minors, since for each *i* we are choosing $k-3$ diagonal elements from $J^{\mathrm {II}}$ and $J^{\mathrm {IV}}$ combined. Using $\sum _{i=0}^{k-3}C_{n-1}^i C_{n-1}^{k-3-i}=C_{2n-2}^{k-3}$, we obtain

$$\begin{aligned} (-1)^{k+1}C_{2n-2}^{k-3}\sum _{i\in {\mathscr {P}}_{1}\left( \{1,2\ldots ,n\}\right) }\psi _i r_i' K\psi _W^k r_G^k \end{aligned}$$

as the total contribution to the right-hand side of Eq. (34). Next, given a pair (*i*, *j*) with $i\ne j$, we consider minors containing diagonal elements from columns *i* and *j* in $J^{\mathrm {II}}$ and columns *i* and *j* in $J^{\mathrm {IV}}$; such a minor contributes $(-1)^{k}r_i' r_j' \psi _i\psi _j K\psi _W^{k-1} r_G^{k-1}$. The multiplicity is $\sum _{i=0}^{k-5}C_{n-2}^i C_{n-2}^{k-5-i}$, since for a given choice (*i*, *j*) there remain $k-5$ elements to be chosen. Summing over all such pairs (*i*, *j*) and using the combinatorics formulae, Eq. (36), we obtain

$$\begin{aligned} (-1)^{k}C_{2n-4}^{k-5}\sum _{(i,j)\in {\mathscr {P}}_{2}\left( \{1,2\ldots ,n\}\right) }r_i' r_j' \psi _i \psi _j K\psi _W^{k-1}r_G^{k-1}. \end{aligned}$$

Next, after pairs of columns, we need to consider triples (*i*, *j*, *k*), and so on. The general formula for an $\ell$-tuple can be obtained via similar reasoning, and summing over all such tuples we obtain

$$\begin{aligned} (-1)^k \sum _{\ell =1}^{n}{\left( C_{2n-2\ell }^{k-(2\ell +1)}K\psi _W^{k+1-\ell }r_G^{k+1-\ell }(-1)^{\ell }\sum _{{\mathbb {S}}\in {\mathscr {P}}_{\ell }\left( \{1,2\ldots ,n\}\right) }\prod _{m\in {\mathbb {S}}}\psi _{m}r_{m}'\right) } \end{aligned}$$

as the final contribution.

Minors of type vi contain contributions from all blocks of the Jacobian matrix, cf. Eq. (33). Each minor contributes $(-1)^k \sum _{i=1}^{k-3}{C_n^i C_n^{k-2-i}K\psi _W^k\left( \psi _W r_G^{k+1}+r_G^{k-1}\right) }$, where the multiplicity arises from the $k-2$ choices from blocks $J^{\mathrm{II}}$ and $J^{\mathrm {IV}}$ combined. In analogy to type iv, minors of type vi contribute the following term $\propto K$:

$$\begin{aligned} (-1)^{k}\sum _{q=1}^{k-3}C_{n}^{q}C_{n-1}^{k-3-q}\sum _{i\in {\mathscr {P}}_{1}\left( \{1,2\ldots ,n\}\right) }r_i'(\sigma _i-r_i\psi _i)K\psi _W^k r_G^{k-1} \end{aligned}$$

and in analogy to type v, minors of type vi contribute the following term $\propto K$:

$$\begin{aligned} (-1)^k \sum _{\ell =1}^{n}{\left( C_{2n-2\ell }^{k-(2\ell +2)}K\psi _W^{k+1-\ell }r_G^{k+1-\ell }(-1)^{\ell }\sum _{{\mathbb {S}}\in {\mathscr {P}}_{\ell }\left( \{1,2\ldots ,n\}\right) }\prod _{m\in {\mathbb {S}}}\psi _{m}r_{m}'\right) }. \end{aligned}$$

There are further terms contributed by minors containing diagonal elements from columns *i* and *j* in block $J^{\mathrm {IV}}$ (where $i\ne j$) in addition to a diagonal element from either columns *i* or *j* in block $J^{\mathrm {II}}$; such minors contribute $(-1)^{k+1}r_i' r_j'\left( \psi _i(\psi _j r_j -\sigma _j)\right.$ $\left. + \psi _j(\psi _i r_i -\sigma _i)\right) K\psi _W^{k-1} r_G^{k-2}$. Summing over all pairs (*i*, *j*) and using the combinatorics formulae, Eq. (36), we obtain

$$\begin{aligned} (-1)^{k+1}C_{2n-3}^{k-5}K\psi _W^{k-1} r_G^{k-2}\sum _{(i,j)\in {\mathscr {P}}_{2}\left( \{1,2\ldots ,n\}\right) }r_i' r_j'\left( \psi _i(\psi _j r_j -\sigma _j) + \psi _j(\psi _i r_i -\sigma _i)\right) . \end{aligned}$$

Next we consider triples (*i*, *j*, *p*), with $i\ne j\ne p$, and minors that take diagonal elements from columns *i*, *j*, and *p* from block $J^{\mathrm {IV}}$ while block $J^{\mathrm{II}}$ contributes diagonal elements from a pair of columns, which is either the pair (*i*, *j*), or (*i*, *p*), or (*j*, *p*). Summing over all triples (*i*, *j*, *p*) and using the combinatorics formulae, Eq. (36), we obtain

$$\begin{aligned}&(-1)^{k}C_{2n-5}^{k-7}\sum _{(i,j,\;p)\in {\mathscr {P}}_{3}\left( \{1,2\ldots ,n\}\right) }r_i' r_j' r_p'\Big (\psi _i\psi _p(\psi _j r_j -\sigma _j)\nonumber \\&\quad + \, \psi _j\psi _p(\psi _i r_i -\sigma _i)+ \psi _i\psi _j(\psi _p r_p -\sigma _p)\Big )K\psi _W^{k-2} r_G^{k-3}. \end{aligned}$$

Generalising this argument to $\ell$-tuples of columns chosen from $J^{\mathrm {IV}}$, we obtain the following:

$$\begin{aligned}&(-1)^k \sum _{\ell =2}^{n}C_{2n-(2\ell -1)}^{k-(2\ell +1)}K\psi _W^{k+1-\ell }r_G^{k-\ell }(-1)^{\ell }\nonumber \\&\quad \times \sum _{{\mathbb {S}}\in {\mathscr {P}}_{\ell }\left( \{1,2\ldots ,n\}\right) }\prod _{m\in {\mathbb {S}}}r_{m}'\left( \sum _{m\in {\mathbb {S}}}(\psi _m r_m -\sigma _m)\prod _{j\in {\mathbb {S}}\setminus m}\psi _j\right) . \end{aligned}$$

A term $(-1)^{k+1}\psi _i r_i' K\psi _W^{k-1} r_G^{k-2}$ is contributed by each minor that takes diagonal elements from column *i* in $J^{\mathrm {II}}$ and from column *i* in $J^{\mathrm {IV}}$. Taking into account the multiplicity $\sum _{i=0}^{k-4}C_{n-1}^i C_{n-1}^{k-4-i}$ and simplifying the sum, we obtain

$$\begin{aligned} (-1)^{k+1}C_{2n-2}^{k-4}\sum _{i\in {\mathscr {P}}_{1}\left( \{1,2\ldots ,n\}\right) }\psi _i r_i' K\psi _W^{k-1} r_G^{k-2}. \end{aligned}$$

For a given pair (*i*, *j*), with $i\ne j$, minors taking diagonal elements from columns *i* and *j* in $J^{\mathrm{II}}$ and from columns *i* and *j* in $J^{\mathrm {IV}}$ contribute $(-1)^{k}r_i' r_j' \psi _i\psi _j K\psi _W^{k-2} r_G^{k-3}$ each. The multiplicity of such minors is $\sum _{i=0}^{k-6}C_{n-2}^i C_{n-2}^{k-6-i}$. Summing over all pairs (*i*, *j*) and simplifying, we have

$$\begin{aligned} (-1)^{k}C_{2n-4}^{k-6}\sum _{(i,j)\in {\mathscr {P}}_{2}\left( \{1,2\ldots ,n\}\right) } r_i' r_j' \psi _i\psi _j K\psi _W^{k-2}r_G^{k-3}. \end{aligned}$$

Generalising to $\ell$-tuples, we obtain

$$\begin{aligned} (-1)^k\sum _{\ell =1}^{n}C_{2n-2\ell }^{k-(2\ell +2)}K\psi _W^{k-\ell }r_G^{k-\ell -1}(-1)^{\ell }\sum _{{\mathbb {S}}\in {\mathscr {P}}_{\ell }\left( \{1,2\ldots ,n\}\right) }\prod _{m\in {\mathbb {S}}}\psi _{m}r_{m}'. \end{aligned}$$

Equation (34) is obtained by collecting terms proportional to *K*. In particular, terms $\propto K\psi _W^{k+1}r_G^{k+1}$ have the following coefficients:

$$\begin{aligned}&(-1)^k C_{n}^{k-1} + (-1)^k C_{n}^{k-1} + (-1)^{k} C_{n}^{k-2} + (-1)^{k} C_{n}^{k-2}\nonumber \\&\qquad + (-1)^k\sum _{i=1}^{k-2}C_{n}^{i}C_{n}^{k-1-i} + (-1)^{k}\sum _{i=1}^{k-3}C_{n}^{i}C_{n}^{k-2-i}\nonumber \\&\quad =(-1)^k\left( \sum _{i=0}^{k-2}C_{n}^{i}C_{n}^{k-1-i} + \sum _{i=0}^{k-3}C_{n}^{i}C_{n}^{k-2-i} + C_{n}^{k-1} + C_{n}^{k-2}\right) \\&\quad =(-1)^k\left( C_{2n}^{k-1} - C_{n}^{k-1} + C_{2n}^{k-2} -C_{n}^{k-2} + C_{n}^{k-1} + C_{n}^{k-2}\right) \\&\quad =(-1)^k\left( C_{2n}^{k-1} + C_{2n}^{k-2}\right) = (-1)^k C_{2n+1}^{k-1}; \end{aligned}$$

terms $\propto K\psi _W^{k}r_G^{k-1}$ have the following coefficients:

$$\begin{aligned}&(-1)^k C_{n}^{k-2} + (-1)^{k} C_{n}^{k-2} + (-1)^{k} \sum _{i=1}^{k-3}C_{n}^{i}C_{n}^{k-2-i}\\&\quad =(-1)^k\left( C_{n}^{k-2} + \sum _{i=0}^{k-3}C_{n}^{i}C_{n}^{k-2-i}\right) \nonumber \\&\quad =(-1)^k\left( C_{n}^{k-2} + C_{2n}^{k-2} - C_{n}^{k-2}\right) = (-1)^k C_{2n}^{k-2}; \end{aligned}$$

terms $\propto K\psi _W^{k+1-\ell }r_G^{k+1-\ell }(-1)^{\ell }\sum _{{\mathbb {S}}\in {\mathscr {P}}_{\ell }\left( \{1,2\ldots ,n\}\right) }\prod _{m\in {\mathbb {S}}}\psi _{m}r_{m}'$ have the following coefficients:

$$\begin{aligned} (-1)^k C_{2n-2\ell }^{k-(2\ell +1)} + (-1)^k C_{2n-2\ell }^{k-(2\ell +2)} = C_{2n-(2\ell -1)}^{k-(2\ell +1)}\quad \text {where }\ \ell \in \{1\dots n\}; \end{aligned}$$

terms $\propto K\psi _W^{k+1-\ell }r_G^{k-\ell }(-1)^{\ell }\sum _{{\mathbb {S}}\in {\mathscr {P}}_{\ell }\left( \{1,2\ldots ,n\}\right) }\prod _{m\in {\mathbb {S}}}r_{m}'\left( \sum _{m\in {\mathbb {S}}}(\psi _m r_m -\sigma _m)\right.$ $\left. \prod _{j\in {\mathbb {S}}\setminus m}\psi _j\right)$ have the following coefficients:

$$\begin{aligned} (-1)^k C_{n-1}^{k-3} + (-1)^k \sum _{q=1}^{k-3}C_{n}^{q}C_{n-1}^{k-3-q}=\sum _{q=0}^{k-3}C_{n}^{q}C_{n-1}^{k-3-q}=C_{2n-1}^{k-3}\quad \text {for }\ \ell =1 \end{aligned}$$

and $$(-1)^k C_{2n-(2\ell -1)}^{k-(2\ell +1)}$$ for $$\ell \in \{2\dots n\}$$.

Recall that $c_k=(-1)^k S_k$ where $S_k$ is the sum of the principal minors $M_k$ that are symmetric with respect to the main diagonal of *J*; thus $S_k$ is a polynomial in *K*. Letting ${\widetilde{S}_k}$ denote the terms in this polynomial that are proportional to *K*, we have ${\widetilde{c}_k}=(-1)^k {\widetilde{S}_k}$ and this implies that

$$\begin{aligned} {\widetilde{c}_k}= & {} (-1)^k (-1)^k\Bigg (C_{2n+1}^{k-1}K\psi _W^{k+1}r_G^{k+1} + C_{2n}^{k-2}K\psi _W^{k}r_G^{k-1}\nonumber \\&+\sum _{\ell =1}^{n}C_{2n-(2\ell -1)}^{k-(2\ell +1)}K\psi _W^{k+1-\ell }r_G^{k-\ell }(-1)^{\ell }\\&\times \sum _{{\mathbb {S}}\in {\mathscr {P}}_{\ell }\left( \{1,2\ldots ,n\}\right) }\prod _{m\in {\mathbb {S}}}r_{m}'\Bigg (r_G\prod _{m\in {\mathbb {S}}}\psi _{m}+\sum _{m\in {\mathbb {S}}}(\psi _m r_m -\sigma _m)\prod _{j\in {\mathbb {S}}\setminus m}\psi _j\Bigg )\nonumber \\&+\sum _{\ell =1}^{n}C_{2n-2\ell }^{k-(2\ell +2)}K\psi _W^{k-\ell }r_G^{k-\ell -1}(-1)^{\ell }\sum _{{\mathbb {S}}\in {\mathscr {P}}_{\ell }\left( \{1,2\ldots ,n\}\right) }\prod _{m\in {\mathbb {S}}}\psi _{m}r_{m}'\Bigg ) \end{aligned}$$

which yields Eq. (34). $\square$

Lemma 1 has a consequence which will prove important in establishing stability of the equilibrium.

##### Corollary 1

(Positive coefficients) The coefficients of the characteristic polynomial, Eq. (32), are all positive for sufficiently large *K*.

##### Proof

The coefficients $c_k$ of the characteristic polynomial, Eq. (32), are first-order polynomials in *K*. Therefore, for *K* is sufficiently large, the sign will agree with that of the terms $\propto K$, since all other terms in $c_k$ are compound expressions made up of the model’s parameters, which are all bounded. It therefore suffices to establish that the ${\widetilde{c}_k}$ are positive. We consider the terms on the right-hand side of Eq. (34) in turn. The first two terms are positive since *K*, $\psi _W$, and $r_G$ are all positive. To determine the sign of the third term we recall that $r_m'<0$ by the assumed properties of $r_m$ as a function of $x_m$ (where $m\in \{1,\dots ,n\}$). Since $m\in {\mathbb {S}}$ and $\mid {\mathbb {S}}\mid = \ell$, we have $\prod _{m\in {\mathbb {S}}}r_m' = (-1)^\ell \prod _{m\in {\mathbb {S}}}\mid {r_m'}\mid$ which means that the third term can be rewritten as follows:

$$\begin{aligned}&\sum _{\ell =1}^{n}C_{2n-(2\ell -1)}^{k-(2\ell +1)}K\psi _W^{k+1-\ell }r_G^{k-\ell }\\&\quad \times \sum _{{\mathbb {S}}\in {\mathscr {P}}_{\ell }\left( \{1,2\ldots ,n\}\right) }\nonumber \prod _{m\in {\mathbb {S}}}\mid r_{m}'\mid \left( r_G\prod _{m\in {\mathbb {S}}}\psi _{m}+\sum _{m\in {\mathbb {S}}}(\psi _m r_m -\sigma _m)\prod _{j\in {\mathbb {S}}\setminus m}\psi _j\right) . \end{aligned}$$

This quantity is positive since $\psi _m r_m-\sigma _m>0$, which can be seen by re-arranging Eq. (29) as

$$\begin{aligned} \psi _m r_m-\sigma _m = \left( 1+x_m\right) \psi _W r_G \end{aligned}$$

and recalling that $x_m\ge 0$ at equilibrium. The final term can be rewritten in analogy to the third term, yielding

$$\begin{aligned} \sum _{\ell =1}^{n}C_{2n-2\ell }^{k-(2\ell +2)}K\psi _W^{k-\ell }r_G^{k-\ell -1}\sum _{{\mathbb {S}}\in {\mathscr {P}}_{\ell }\left( \{1,2\ldots ,n\}\right) }\prod _{m\in {\mathbb {S}}}\psi _{m}\mid r_{m}'\mid , \end{aligned}$$

which is positive for $m\in \{1,2,\ldots ,n\}$. Thus, all terms in Eq. (34) are positive and therefore ${\widetilde{c}_k}>0$ for $k\in \{1,2,\ldots ,2n+2\}$. It follows that the coefficients of the characteristic polynomial, Eq. (32), are all positive for sufficiently large *K*.$\square$

#### Stability of the characteristic equation

##### Theorem 1

(Strelitz) A monic polynomial with real coefficients is stable if and only if the coefficients of both this polynomial and its sum-of-roots polynomial are all positive.

We omit the proof, which can be found in Strelitz (1977), who also shows how to compute the coefficients of the sum-of-roots polynomial efficiently in terms of power-sums. This algorithm exploits the Newton-Girard formulae for power-sums of polynomial roots (Séroul 2000) and Strelitz’s recurrence formula. Let

$$\begin{aligned} f=\lambda ^{2n+2} +{\widetilde{c}_1}\lambda ^{2n+1} + {\widetilde{c}_2}\lambda ^{2n} + \dots + {\widetilde{c}_{2n+1}}\lambda + {\widetilde{c}_{2n+2}}, \end{aligned}$$

where the coefficients ${\widetilde{c}_i},i\in \{1,2,\ldots ,2n+2\},$ are given by Eq. (34). The sum-of-roots polynomial is

$$\begin{aligned} g=\lambda ^{m} +b_1\lambda ^{m-1} + b_2\lambda ^{m-2} + \dots + b_{m-1}\lambda + b_{m}, \end{aligned}$$

where $m=(2n+2)(2n+1)/2=(n+1)(2n+1)$ and the $b_m$ are to be determined by means of Strelitz’s algorithm. Let $\{\alpha _i\}_{i\in \{1,\ldots ,2n+2\}}$ be the set of all $2n+2$ roots of *f*, and $\{\beta _i\}_{i\in \{1,\ldots ,m\}}=\{\alpha _i +\alpha _j\}_{1\le i<j\le 2n+2}$ be the set of all *m* roots of *g*. The power sums of *f* and *g* will be denoted as

$$\begin{aligned} \zeta _j=\sum _{i=1}^{2n+2}\alpha _i^j,\quad s_j=\sum _{i=1}^{m}\beta _i^j,\quad j\in \{0,1,\ldots ,m\}, \end{aligned}$$

respectively. Strelitz’s algorithm consists of three main steps:

Step 1 Express $\zeta _j$ in terms of ${\widetilde{c}_i}$, where $i\in \{0,1,\ldots 2n+2\},\; j\in \{0,1,\ldots ,m\}$.
Step 2 For $k\in \{0,1,\ldots ,m\}$, express $s_k$ in terms of ${\widetilde{c}_i}$, where $i\in \{0,1,\ldots 2n+2\}$, via the $\zeta _j$, $j\in \{0,1,\ldots ,m\}$, which are obtained in Step 1.
Step 3 For $\ell \in \{0,1,\ldots ,m\}$, express $b_{\ell }$ in terms of ${\widetilde{c}_i}$, where $i\in \{0,1,\ldots 2n+2\}$, via the $s_k$, $k\in \{0,1,\ldots ,m\}$, which are obtained in Step 2.

Step 1 relies on the Newton-Girard formulae:

37 $$\begin{aligned}&\zeta _0 =2n+2\nonumber \\&\zeta _1 + {\widetilde{c}_1}=0\nonumber \\&\zeta _2+\zeta _1{\widetilde{c}_1} + 2{\widetilde{c}_2}=0\nonumber \\&\vdots \nonumber \\&\zeta _{j}+\zeta _{j-1}{\widetilde{c}_1} + 2\zeta _{j-2}{\widetilde{c}_2} +\cdots + j{\widetilde{c}_j}=0\nonumber \\&\vdots \nonumber \\&\zeta _{2n+2}+\zeta _{2n+1}{\widetilde{c}_1} + 2\zeta _{2n}{\widetilde{c}_2} +\cdots + (2n+2){\widetilde{c}_{2n+2}}=0 \end{aligned}$$

where for $j>2n+2$ we have

38 $$\begin{aligned} \zeta _j + \zeta _{j-1}{\widetilde{c}_1} +\cdots + \zeta _{j-(2n+2)}{\widetilde{c}_{2n+2}}=0. \end{aligned}$$

We shall write $\bar{c_i}={\widetilde{c}_i}/K$, so that ${\widetilde{c}_i}=K\bar{c_i}$. From system (37) we find the $\zeta _j$:

39 $$\begin{aligned} \zeta _0= & {} 2n+2\nonumber \\ \zeta _1= & {} - {\widetilde{c}_1} = -K\bar{c_1}\nonumber \\ \zeta _2= & {} -\zeta _1{\widetilde{c}_1} - 2{\widetilde{c}_2} = K^2\bar{c_1}^2 - 2K\bar{c_2}\nonumber \\ \zeta _3= & {} -\zeta _2{\widetilde{c}_1} - 2\zeta _1{\widetilde{c}_2} - 3{\widetilde{c}_3} = -K^3\bar{c_1}^3 + 2K^2\bar{c_1}\bar{c_2} - 3K\bar{c_3}\nonumber \\ \vdots&\end{aligned}$$

which shows that the $\zeta _j$ are polynomials in *K*. Let ${\widetilde{\zeta }_j}$ denote the term in the polynomial $\zeta _j$ with the leading power of *K*. If *K* is sufficiently large, we need only consider these polynomials to leading power of *K*, since the $\bar{c_i},\;i\in \{1,2,\ldots ,2n+2\}$, consist of model parameters, each of which is bounded.

##### Lemma 2

For each polynomial $\zeta _j$, where $j\in \{2,3,\ldots ,m\}$ only the term $-\zeta _{j-1}{\widetilde{c}_1}$ contributes to the leading power of *K*, in particular,

40 $$\begin{aligned} {\widetilde{\zeta }_j} = (-1)^j K^j \bar{c_1}^j. \end{aligned}$$

We remark that the case $j=1$ is irrelevant since $\zeta _1$ does not depend on $\zeta _0$.

##### Proof

The proof is by induction. For $j=2$ we have, from system (39):

$$\begin{aligned} \zeta _2= -\zeta _1{\widetilde{c}_1} - 2{\widetilde{c}_2} = K^2\bar{c_1}^2 - 2K\bar{c_2}, \end{aligned}$$

so that ${\widetilde{\zeta }_2} = K^2\bar{c_1}^2$ which agrees with the claim, since it is derived from $-\zeta _1{\widetilde{c}_1}$ and conforms to the formula $(-1)^j K^j \bar{c_1}^j$, Eq. (40). For the induction step, we assume that Eq. (40) holds for $j=k$, i.e., the term with leading power of *K* of the polynomial $\zeta _k$ is contributed by $-\zeta _{k-1}{\widetilde{c}_1}$ which is the first term in the general form

41 $$\begin{aligned} \zeta _{k}= -\zeta _{k-1}{\widetilde{c}_1} - 2\zeta _{k-2}{\widetilde{c}_2} - \cdots - k{\widetilde{c}_k} \end{aligned}$$

and, moreover, it is true that

42 $$\begin{aligned} {\widetilde{\zeta }_k} = (-1)^k K^{k}\bar{c_1}^k. \end{aligned}$$

By the induction hypothesis, $\zeta _{k-1}$ contributes a term $\propto K^{k-1}$ and all terms other than the first in the right-hand side of Eq. (41) must contribute powers of *K* less than *k*, which in turn implies that $\zeta _{k-2},\;\zeta _{k-3},\;\ldots ,\;\zeta _{1}$ contribute powers of *K* less than $k-1$ as ${\widetilde{c}_i}=K\bar{c_i}$ for $i\in \{1,2,\ldots ,2n+2\}$. To prove that the claim is correct for $j=k+1$ we consider the polynomial $\zeta _{k+1}$, which by the Newton-Girard recursion takes on the following form:

43 $$\begin{aligned} \zeta _{k+1}= -\zeta _{k}{\widetilde{c}_1} - 2\zeta _{k-1}{\widetilde{c}_2} - \cdots - (k+1){\widetilde{c}_{k+1}}. \end{aligned}$$

By induction hypothesis, $\zeta _{k}$ contributes the leading power *k* of *K*, while $\zeta _{k-1}$ contributes $K^{k-1}$ and $\zeta _{k-2}$, $\zeta _{k-3}, \ldots , \zeta _{1}$ contribute powers of *K* less than $k-1$. Thus, the leading-power term in $\zeta _{k+1}$ can only be $-\zeta _{k}{\widetilde{c}_1}$. Since $-\zeta _{k}{\widetilde{c}_1} = -\zeta _{k} K\bar{c_1}$, the leading term is $-{\widetilde{\zeta }_k} K\bar{c_1}$, which can be combined with Eq. (42) to give ${\widetilde{\zeta }_{k+1}}= (-1)^{k+1} K^{k+1}\bar{c_1}^{k+1}$ which agrees with Eq. (40) for $j=k+1$, as required. An entirely analogous argument using Eq. (38) establishes the result for $j>2n+2$.$\square$

As we have $\zeta _1= -K\bar{c_1}$ from Eq. (39), we can claim that

44 $$\begin{aligned} {\widetilde{\zeta }_0}=2n+2\,\text {and}\, {\widetilde{\zeta }_j}= (-1)^{j} K^{j}\bar{c_1}^{j}\quad \text {for }\ j=1,2,\ldots ,m. \end{aligned}$$

We proceed to Step 2 of Strelitz’s algorithm, expressing $s_k$ in terms of ${\widetilde{c}_i}$ by means of the formulae for ${\widetilde{\zeta }_j}$ which were obtained in the previous step. Strelitz’s recurrence formulae relate power-sums for *g* to those of *f*:

$$\begin{aligned} 2 s_j = \sum _{p=0}^{j}C_{j}^{p}\zeta _{p}\zeta _{j-p} - 2^j\zeta _{j},\quad j\in \{0,1,\ldots ,m\}. \end{aligned}$$

As $\zeta _j$ are polynomials in *K*, the $s_j$ are also polynomials in *K*. Letting ${\widetilde{s}_j}$ denote the term in the polynomial $s_j$ with leading power of *K*, we have

$$\begin{aligned} 2 {\widetilde{s}_j} = \sum _{p=0}^{j}C_{j}^{p}{\widetilde{\zeta }_p}{\widetilde{\zeta }_{j-p}} - 2^j{\widetilde{\zeta }_j},\qquad j\in \{0,1,\ldots ,m\}. \end{aligned}$$

By system (44), we have

$$\begin{aligned} 2 {\widetilde{s}_j}&= C_{j}^{0}{\widetilde{\zeta }_0}{\widetilde{\zeta }_j} +C_{j}^{j}{\widetilde{\zeta }_j}{\widetilde{\zeta }_0} + \sum _{p=1}^{j-1}C_{j}^{p}{\widetilde{\zeta }_p}{\widetilde{\zeta }_{j-p}} - 2^j{\widetilde{\zeta }_j}\nonumber \\&=2 (2n+2) (-1)^{j} K^{j}\bar{c_1}^{j} + \sum _{p=1}^{j-1}C_{j}^{p} (-1)^{j} K^{j}\bar{c_1}^{j} - 2^j (-1)^{j} K^{j}\bar{c_1}^{j}\\&=2 (2n+2) (-1)^{j} K^{j}\bar{c_1}^{j} + (-1)^{j} K^{j}\bar{c_1}^{j}\left( \sum _{p=1}^{j-1}C_{j}^{p} - 2^j\right) . \end{aligned}$$

The term between brackets can be written as $\sum _{p=0}^{j}C_{j}^{p} - C_{j}^{0} - C_{j}^{j} - 2^j$ which can be simplified by means of the combinatoric equation $\sum _{p=0}^{j}C_{j}^{p} = 2^j$, yielding:

45 $$\begin{aligned} {\widetilde{s}_j} = (-1)^{j} K^{j}\bar{c_1}^{j}(2n+1),\quad j\in \{1,2\dots ,m\}. \end{aligned}$$

To express the $b_\ell$ in terms of the ${\widetilde{c}_i}$ via the ${\widetilde{s}_j}$, we again use the Newton-Girard formulae, replacing *m*, $b_\ell$, and ${\widetilde{s}_j}$ by *n*, ${\widetilde{c}_i}$, and $\zeta _j$, respectively in system (37), which gives

46 $$\begin{aligned}&{\widetilde{s}_0}=m\nonumber \\&{\widetilde{s}_1} + b_1=0\nonumber \\&{\widetilde{s}_2} + {\widetilde{s}_1} b_1 + 2b_2=0\nonumber \\&\vdots \nonumber \\&{\widetilde{s}_{\ell }} + {\widetilde{s}_{\ell -1}} b_1 + 2{\widetilde{s}_{\ell -2}} b_2 +\cdots + \ell b_{\ell }=0\nonumber \\&\vdots \nonumber \\&{\widetilde{s}_m}+{\widetilde{s}_{m-1}} b_1 + 2{\widetilde{s}_{m-2}} b_2 +\cdots + m b_{m}=0 \end{aligned}$$

By Eq. (45) we have

47 $$\begin{aligned}&b_1= - {\widetilde{s}_1} = K\bar{c_1}(2n+1)\nonumber \\&2b_2= -{\widetilde{s}_2} - {\widetilde{s}_1} b_1 = K^2\bar{c_1}^2(2n+1)2n\nonumber \\&3b_3= -{\widetilde{s}_3} - {\widetilde{s}_2} b_1 - 2{\widetilde{s}_1 }b_2 = K^3\bar{c_1}^3(2n+1)(2n)^2\nonumber \\&\vdots \end{aligned}$$

The pattern that is apparent from these first few terms holds in general, as asserted by the following lemma.

##### Lemma 3

Each coefficient $b_\ell$ is given by the following formula

48 $$\begin{aligned} b_\ell = \frac{1}{\ell } K^{\ell } \bar{c_1}^{\ell }(2n+1)(2n)^{\ell -1},\quad \ell \in \{1,2,\ldots ,m\}. \end{aligned}$$

##### Proof

The proof is by induction. The case $\ell =1$ is immediate since the first equation of system (47) is of the form claimed. For the induction step, we assume that Eq. (48) is correct for $\ell =k$, which implies

49 $$\begin{aligned} b_k = \frac{1}{k} K^{k} \bar{c_1}^{k}(2n+1)(2n)^{k-1}. \end{aligned}$$

It follows from Eq. (46) that

$$\begin{aligned} k b_{k} = -{\widetilde{s}_k} - {\widetilde{s}_{k-1}} b_1 - 2{\widetilde{s}_{k-2}} b_2 -\cdots - (k-1){\widetilde{s}_1} b_{k-1}, \end{aligned}$$

which means that

50 $$\begin{aligned} -{\widetilde{s}_k} - {\widetilde{s}_{k-1}} b_1 - 2{\widetilde{s}_{k-2}} b_2 -\cdots - (k-1){\widetilde{s}_1} b_{k-1} = K^{k} \bar{c_1}^{k}(2n+1)(2n)^{k-1}\qquad \end{aligned}$$

by hypothesis, Eq. (49). Multiplying both sides of Eq. (50) by $(-1)^{k+1}K\bar{c_1}$, we have, with Eq. (45):

$$\begin{aligned}&(-1)^{k+1}\left( {\widetilde{s}_{k+1}} + {\widetilde{s}_k} b_1 + 2{\widetilde{s}_{k-1}} b_2 + \cdots + (k-1){\widetilde{s}_2} b_{k-1}\right) \\&\quad = (-1)^{k+1}K^{k+1} \bar{c_1}^{k+1}(2n+1)(2n)^{k-1}, \end{aligned}$$

whose left-hand side can be rewritten as $(-1)^{k+1}\left( -(k+1)b_{k+1}-k{\widetilde{s}_1 }b_k\right)$ by system (46), giving

$$\begin{aligned} -(k+1)b_{k+1}-k{\widetilde{s}_1 }b_k = K^{k+1} \bar{c_1}^{k+1}(2n+1)(2n)^{k-1}, \end{aligned}$$

which yields

$$\begin{aligned} -(k+1)b_{k+1}&= k{\widetilde{s}_1 }b_k + K^{k+1} \bar{c_1}^{k+1}(2n+1)(2n)^{k-1}\\&= - k K\bar{c_1}(2n+1)b_k + K^{k+1} \bar{c_1}^{k+1}(2n+1)(2n)^{k-1}, \end{aligned}$$

whence

$$\begin{aligned} (k+1)b_{k+1}&= k K\bar{c_1}(2n+1){k}^{-1} K^{k} \bar{c_1}^{k}(2n+1)(2n)^{k-1}\\&\quad - K^{k+1} \bar{c_1}^{k+1}(2n+1)(2n)^{k-1}\\&= K^{k+1} \bar{c_1}^{k+1}(2n+1)^2(2n)^{k-1} - K^{k+1} \bar{c_1}^{k+1}(2n+1)(2n)^{k-1}\\&= K^{k+1} \bar{c_1}^{k+1}(2n+1)(2n)^{k-1}\left( 2n+1 - 1\right) \\&= K^{k+1} \bar{c_1}^{k+1}(2n+1)(2n)^{k}, \end{aligned}$$

which agrees with Eq. (48) for $\ell =k+1$, as required. $\square$

##### Corollary 2

The coefficients of the sum-of-roots polynomial of the characteristic polynomial, Eq. (32), are all positive for sufficiently large *K*.

##### Proof

By Lemma 2, the coefficients of the sum-of-roots polynomial of the characteristic polynomial for sufficiently large *K* are given by

$$\begin{aligned} b_\ell = \frac{1}{\ell } K^{\ell } \bar{c_1}^{\ell }(2n+1)(2n)^{\ell -1}\quad \ell \in \{1,2,\ldots ,m\}, \end{aligned}$$

where $\bar{c_1} = {\widetilde{c}_1}/K = \psi _W^2r_G^2 > 0$ by Lemma 1. Since $\ell$, *n*, and *K* are all positive, we conclude that $b_\ell >0$ for $\ell \in \{1,2,\ldots ,m\}$. $\square$

The stability of the equilibrium point of system (8) has now been established for sufficiently large *K*, since the characteristic polynomial is stable. This follows from Theorem 1, together with Corollaries 1 and 2, which show that the coefficients are all positive and real.

## Discussion

We have presented and analysed an extension of a VIS-type model for microbial growth and metabolism, explicitly accounting for the dynamic allocation of cellular resources over various types of catalytic machinery. The analysis suggests that dynamic allocation is central to adaptive responses to changing environmental conditions. This allocation can be charted in detail as a function of time through experimental approaches such as ribosome profiling and detailed proteomics, recent developments that prompt an extension of the VIS theory to incorporate such data.

The general approach is modular: our categorisation of machinery is comparatively coarse-grained, e.g., collecting all proteins involved in the uptake of glucose into a single component, an assumption bolstered by the “proportional synthesis” principle (Li et al. 2014). However, the set-up of the model readily lends itself to a more fine-grained treatment.

Whereas the stoichiometric part of the theory relies on basic conservation principles and hence ought to be uncontroversial, the constitutive relations are more speculative. It is therefore important to emphasise that the latter can be reconstructed from observational data.

We closed the dynamics via the *r*-functions and a normalisation corresponding, broadly speaking, to the relative amount of ‘ribosome time’ devoted to the manufacture of the various types of machinery. These *r*-functions express the respective propensities for the various types of machinery to be synthesised, and, in somewhat anthropomorphic terms, indicate how urgently the cell requires the various types. The formalism presented here shares a mechanism of regulation of growth via machinery-making machinery (ribosomes) with the Scott-Hwa-model (Scott et al. 2010, 2014; Scott and Hwa 2011); another point of agreement is the effective implementation of building block allocation via ‘ribosome time.’ In both cases, the formalisms are in keeping with well-established knowledge of microbial physiology; in particular, the steady-state relationship between RNA and growth rate (Herbert 1961) is fundamental to the feedback mechanism expressed here by Eq. (11).

Even if the modelling of allocation variables via *r*-functions is rejected, there is merit in the general approach of reconstructing a ‘regulatory map’ from the state $\{m_0,\dots ,m_G,x_1,\dots , x_n\}$. Provided that the numerical values of the stoichiometric coefficients can be determined, this map can be recovered from data on cell quota combined with bioproduction rates. Moreover, we anticipate that additional information can be gleaned by studying data obtained from step changes in environmental conditions imposed on a continuous-growth culture. Regulatory maps can thus be reconstructed from data obtained under a broad range of environmental conditions.

If these maps agree, this would instil confidence in this simple approach, and if not, more complex models are required, for instance incorporating additional state variables (e.g. signalling machinery, epigenetic status) as well as direct environmental input on gene expression, which in prokaryotes is primarily mediated by two-component systems (Whitworth and Cock 2008). It is not *a priori* obvious how a cell might integrate feedforward (signals emanating from ambient conditions) and feedback (signals from internal status such as reserves) regulation. Ambient stimuli could be the main driver, with a modulatory role for reserve status. Alternatively, reserves transmit a message effectively expressing the urgency of requirements for certain building blocks, whilst the ambient signals are used to decide between alternative sources to replenish these reserves, that is, where the organism is capable switching between, e.g., alternative carbon sources, it would dispose of the genetic material encoding the assimilatory machineries that can handle these respective alternative nutrients, and feedforward-type signals could be key to driving changes in gene expression, corresponding in our formalism by the *r*-factors.

The shape of the function used to relate a reserve density to an *r*-factor dictates whether this reserve is subject to stringent homeostasis, or whether it is allowed to wax and wane along with changing nutrient availability, as demonstrated in qualitative terms in Sect. 4.3 (the mid-point slope parameter is particularly important in this respect). If homeostasis is stringent for all reserve densities, a strong version of balanced growth ensues, as the overall biomass composition is also kept constant or at least maintained within narrow margins of variation. It can be shown that this type of regulation maximises the specific growth rate $\mu$ (van den Berg et al. 2002) but it would be a mistake to equate $\mu$ to fitness outright, as has been done in the past (Kompala et al. 1984; Lenski et al. 1991); only under certain, quite restrictive, conditions on the manner in which the environment varies and on the types of competitors faced by the organism, does $\mu$ agree with the correct general expression for fitness (van den Berg et al. 2008; Metz et al. 1992). Thus, under different ecological circumstances, such as, for instance, a regular alteration of the availability of carbon and nitrogen sources, different reserve management strategies will be favoured by natural selection (Parnas and Cohen 1976).

We have represented nutrient influx through the assimilatory machinery $M_i$ by a term of the form ${\widehat{\widetilde{\phi _i}}} f_i M_i$, where ${\widehat{\widetilde{\phi _i}}}$ represents a maximum possible influx and $f_i$ could be, for instance, the Michaelis-Menten hyperbola, or some other rational function according to the details of the work cycle of the uptake machinery (van den Berg 2011). This implies that a two-fold reduction in $f_i$ can be compensated by a two-fold increase in $M_i$, which as we have seen is key to the attainability of balanced growth. Light-harvesting in photosynthetic bacteria provides a dramatic demonstration of the ability of increased $M_i$ to compensate for low $f_i$ (Beveridge 1989). However, if there is an unstirred layer around the bacterium, the probability that a nutrient particle that has reached the cell wall will diffuse toward one of the nutrient-uptake pores before returning to the bulk phase must be taken into account. This probability increases asymptotically towards 1 as the pore density (which is $\propto M_i$) increases, which means that a less than proportional increase in the total flux is accomplished by increasing $M_i$ (Berg and Purcell 1977). Thus $f_i$ is a function of both the bulk phase concentration of the nutrient and of $M_i$. The ‘idle time fraction’ $1-f_i$ could therefore serve as a signal that is carried back to the genome, to modulate the expression of genes encoding the $M_i$-machinery.

According to the equations of Sect. 2.1, the distribution of building blocks among the machinery relaxes to that imposed by the allocation constants with a time constant of order $\mu ^{-1}$. This may be too slow for reserves where the organism cannot tolerate large excursions, as will be the case when the reserve is physically represented by a small metabolite which is chemically reactive, or when the reserve consists of a chemical moiety as part of a regenerative cycle, which limits the capacity of storage (Reitzer 2003). By contrast, reserves that occur as polymers or elemental crystals, often stored as cellular inclusions, tend to be chemically inert and large variations in the fraction of cellular dry weight they represent can be withstood without affecting the function of core metabolism (Neidhardt et al. 1990). More rapid-acting pathways act to regulate such critical narrow-range or narrow-capacity reserves; on the top-end, an additional feedback to the influx is needed. The nutrient influx formula ${\widehat{\widetilde{\phi _i}}} f_i M_i$ is then extended with an additional multiplier, close to 1 when the reserve density remains below the critical maximum or capacity and steeply decreasing to 0 when the reserve approaches the critical value. Such an additional multiplier is a natural way to represent the chemical-kinetic interactions that modulate the efficacy of such systems (Deutscher et al. 2014; Hariharan et al. 2015; Reitzer 2003). As reserves approach critical depletion, the organism may switch to a different metabolic mode, or slow down the overall rate of metabolism to suit the diminished supply (van den Berg 1998a). We anticipate that this can be brought into the present theory by introducing “sliding dynamics” to prevent reserve densities from assuming negative values.

At the microscopic level, biochemical reaction rates are governed by concentrations of both reactants or products, or possibly only by those of the reactants, but not by the products alone—lest reactant concentrations can become negative, which is physically impossible. Yet in the equations of the present theory, which represent the organisation of fluxes at the macro-chemical level, such reactant-control (supply-side or donor-control) is lacking, limiting the validity of the model to those situations where sufficient reserves are left to replenish the pools of core metabolites. Negative reserve densities are tantamount to a breach of the homeostasis of the structural component, as these core metabolites are depleted.

Metabolic slow-down (or even shut-down) is related to the energy charge of the cell (van den Berg 1998b), which can be conceptualised as a store of ‘phosphorylation equivalents’ (PEs) which are physically realised as phosphate moieties on purines (Dawes 1989; Nicholls and Ferguson 1992). Numerous cellular processes rely on the availability of PEs to proceed (Neidhardt et al. 1990), and as a consequence the range of tolerable variation is narrow, with end points that are guarded by rapid-acting processes. At the upper end of the range, these processes include the use of ATP to generate polyphosphate reserves (Preiss 1989), as well as a reduction of the efficiency of the processes that generate proton-motive force—these may be driven by light-harvesting, or electron-transfer chains coupled to oxidation and reduction of external substrates; in the latter case the modulation is known as “uncoupling” (Nicholls and Ferguson 1992). When the energy charge is low, these changes are reversed (redox coupling or light harvesting efficiency is maximised) and if this does not suffice, reserves such as polyphosphate, glucans, lipids, and poly-$\beta$-hydroxybutyrate are mobilised (Dawes 1989). This endogenous reserve mobilisation flux is one for which we should represent the dependence on the donor (the reserve) explicitly. If, despite maximal up-modulation stimulated by the low energy charge, this donor limitation limits the mobilisation flux to below the level required to replenish the PE pool, the latter is gradually depleted, and these critically low levels are coupled to a reduction in all rates (van den Berg 1998a; Dawes 1989).

The cellular management of the energy charge has not been represented explicitly in the present paper; such an extension is necessary to accommodate the processes that happen at zero (or very low) specific growth rates and starvation. Virtually all rate terms that figure explicitly in the present paper are dependent on PEs; to the list of energy requirements should be added maintenance requirements (Marr et al. 1962; Dawes 1989) which have been ignored here for the sake of simplicity.

Reducing equivalents (REs) mediate coupling between energy and nutrient budgets and serve as a ‘co-nutrient’ when the external growth substrate must be reduced to form cellular constituents (Nicholls and Ferguson 1992). These REs physically exist as ($\mathrm{e}^{-}$, $\mathrm{H}^{+}$) pairs that are carried by specialised co-enzymes which in reduced form carry 1 RE and can transfer it on as they are oxidised (Nicholls and Ferguson 1992). REs can be derived from the oxidation of organic compounds, this being the sole source in organotrophs, or from the oxidation of inorganic substrates (in lithotrophs) (Neidhardt et al. 1990). REs are expended in respiration (reducing an inorganic substrate, called the terminal electron acceptor, via a chain of redox transfers of the REs, generating proton-motive force in the process), in fermentation or disproportionation (using organic compounds as electron acceptors), and (in autotrophs) to reduce the carbon in inorganic nutrients to the levels of the organic building blocks required for biosynthesis (Neidhardt et al. 1990). Not all of these sources and sinks occur simultaneously in every bacterium, although some organisms are remarkably versatile (van Gemerden 1993; Schaub and Gemerden 1994).

The status of the RE pool is primarily governed by its kinetic coupling to physiological fluxes that require or produce REs, as well as environmental availabilities of light and redox substrates. However, only when a terminal electron acceptor is not available for respiration, are REs expended on the endogenous reduction of organic compounds in fermentation, which is energetically less favourable than respiration; this is the Pasteur effect (Neidhardt et al. 1990). Furthermore, the degradation of poly-$\beta$-hydroxybutyrate, a source of REs, is inhibited, provided there are exogenous carbon sources (Preiss 1989). A related conditional redox mode switch ensures the avoidance of dissipative idle cycles; for instance, in photo- and litho-autotrophs formation of energy reserves requires an investment of REs and PEs which is at best recovered without net gain when these reserves are concomitantly used to replenish the energy charge. Thus in the absence of exogenous electron donors and/or light, such assimilatory processes should be halted (van den Berg 1998b). This can be modelled by regarding these assimilatory fluxes to have $f_i$-factors which depend on the availabilities of both the building-block substrate and the energy-yielding substrate.
